# Pathological Ossification of Spinal Ligament‐Derived Fibroblasts in an Inflammatory Micro‐Environment via Activation of the ECM/ITGA5/FAK/AKT Pathway in Ankylosing Spondylitis

**DOI:** 10.1111/cpr.70299

**Published:** 2026-11-06

**Authors:** Mu Qiao, Kai‐Yang Wang, Chen‐Yu Song, Jing‐Shun Lu, Jiu‐Kun Li, Yong Qiu, Bang‐Ping Qian, Ze‐Zhang Zhu

**Affiliations:** ^1^ Division of Spine Surgery, Department of Orthopedic Surgery Nanjing Drum Tower Hospital, Affiliated Hospital of Medical School, Nanjing University Nanjing China; ^2^ Department of Orthopedic Surgery Shanghai Sixth People's Hospital Affiliated to Shanghai Jiao Tong University School of Medicine Shanghai China; ^3^ Department of Orthopedic Surgery Yijishan Hospital of Wannan Medical College Wuhu Anhui China

**Keywords:** ankylosing spondylitis, fibroblast, inflammatory micro‐environment, ITGA5, pathological osteogenesis

## Abstract

Chronic inflammation and heterotopic ossification (HO) were typical hallmarks of ankylosing spondylitis (AS). Although existing target inflammation inhibitors could reduce inflammation but failed to halt HO progression, highlighting the need for osteogenesis‐specific treatments. However, the role of fibroblasts (Fbs) responsible for extracellular matrix (ECM) remodelling in AS‐associated pathological ossification remained unclear. In this study, osteogenic capacity, pathological condition and underlying molecular pathways for Fbs derived from AS spinal ligaments were validated. Fbs from uncalcified spinal ligaments of age‐matched AS patients/controls were compared. AS‐Fbs were treated with a gradient concentration of TNF‐α during osteogenic induction. Meanwhile, specific inhibition of AKT phosphorylation was applied to confirm the role of PI3K/AKT in osteogenesis. Upstream signalling pathways were also explored via RNA‐sequencing. Additionally, in vivo, subcutaneous cell transplantation was performed to simulate ectopic ossification. AS‐Fbs possessed superior osteogenic capacity than controls. Moreover, osteogenic signs could be promoted by low concentration TNF‐α‐induced inflammatory micro‐environment. Functional analysis revealed a crucial role of the PI3K/AKT pathway. Mechanistically, RNA‐seq identified the ECM/integrin subunit alpha 5 (ITGA5)/focal adhesion kinase (FAK) upstream pathway. In detail, osteogenic induction increased ECM protein levels including thrombospondin‐1 (THBS1) and fibulin‐1 (FBLN1), activating ITGA5, subsequent FAK phosphorylation, osteogenic markers expression and eventually inducing Fb‐to‐osteocyte differentiation. ITGA5 knockdown or specific FAK inhibition attenuated osteogenic phenotypes and reduced osteogenic proteins expression. Regarding in vivo experiments, subcutaneous bone‐like tissue formation derived from transplantation of AS‐Fbs was identified and could be suppressed significantly via the AKT phosphorylation inhibition. In conclusion, the findings of this study revealed potential causes for HO progression derived from AS‐Fbs in spite of effective anti‐inflammation therapy. Furthermore, the crucial role of ECM/ITGA5/FAK/AKT axial signalling pathways in the aberrant osteogenesis of AS‐Fbs was identified. Combined targeted intervention at the aforementioned signals and application of anti‐inflammatory agents might be a novel potential strategy for the treatment of pathological ectopic ossification caused by AS.

## Graphical abstract

Schematic diagram illustrating the activation of the ECM/ITGA5/FAK/AKT axial signalling pathway in the osteogenic transdifferentiation of AS ligaments‐derived fibroblasts and the simulation of ectopic ossification via subcutaneous transplantation in vivo.

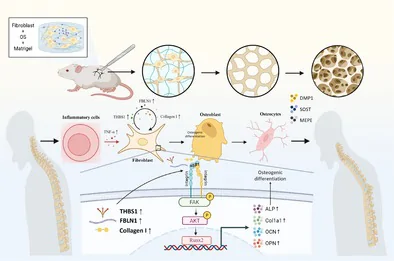

## 1 Introduction

Ankylosing spondylitis (AS) was a chronic, autoimmune, inflammatory disease that primarily affected the axial skeleton [1, 2]. In the advanced stage, progressive structural destruction and stiffness of the entire spine might lead to restricted mobility, cosmetic and functional impairments. Chronic inflammation and heterotopic ossification (HO), the hallmarks of AS, have long been the focus of researches [3, 4, 5]. Significant advances have been reported in the literature regarding interventions and treatments targeting inflammation in AS [6, 7, 8, 9, 10]. Yet it was worth noting that even if the TNF‐α inhibitors, IL‐17A blockers, or Janus kinase (JAK) inhibitors could effectively reduce the inflammation and secondary pain, these therapies failed to halt the HO progression [11, 12, 13, 14]. This paradoxical interplay between inflammatory changes and pathological bone formation was central to the progression of AS, which was the key issue that warranted exploration in this study.

With regard to the discrepancy of inflammation control and HO intervention, this study relied on the following multiple hypotheses as the basis for conducting related studies. First of all, the chronic inflammatory state in AS was mediated by a variety of distinct cells and signalling pathways, including immune cell infiltration (e.g., Th17 cells), pro‐inflammatory cytokines (e.g., TNF‐α, IL‐17A), and the JAK/STAT and NF‐ᴋB pathways. Consequently, the degree of inflammatory control achieved following anti‐inflammatory drug administration varied substantially among different individuals. Whether the residual inflammatory status or inflammatory microenvironment still exerted any inductive or stimulatory effects on target cell populations remained to be further verified. In addition, emerging evidence highlighted fibroblasts (Fbs)–also originated from the embryonic mesenchymal cells like Bone Marrow Mesenchymal Stem Cell (BMSC)–as one of the key contributors to the initiation and development of pathological ossification [15, 16, 17, 18]. Thus, Fbs as the principal stromal cells in ligaments were identified as the subject of the current research, which demonstrated the unique phenotypic plasticity and osteogenic potential according to the previous literature [15, 16, 19].

Second, the underlying molecular mechanisms for inflammation and ossification might exhibit partially independent pathways. Anti‐inflammatory therapies failed to directly target or fully cover the key drivers of pathological osteogenesis for AS‐Fbs, leading to difficulties in clinical treatment for persistent ectopic ossification progression. Activation of multiple pathways has been reported to be associated with the osteogenic differentiation of hip joint capsules‐derived Fbs in AS, including Wnt/β‐catenin, BMPs/Smad, etc. [20, 21, 22, 23]. However, in terms of the spinal ligaments‐derived Fbs, it was of great importance to determine other specific signalling pathways involved in the transdifferentiation process to define novel therapeutic targets.

Therefore, this study was proposed to elucidate the reaction of Fbs‐associated osteogenesis to various pathological stimuli and micro‐environment conditions, to identify the underlying axial signalling pathways, thereby identifying more targets for HO suppression in combination with anti‐inflammatory therapy.

## 2 Materials and Methods

### 2.1 Ethics Approval and Consent to Participate

Informed consent was obtained from all patients for being included in the study. Ethics Committee of Nanjing drum tower Hospital agreed to this experiment (2024‐787‐03). All the animal experiments were reviewed and approved by the Laboratory Animal Ethics Committee of Nanjing Hospital Affiliated to Nanjing Medical University and Nanjing Drum Tower Hospital, Affiliated Hospital of Medical School, Nanjing University (DWSY‐23035339). The raw data used or analyzed in the study were available from the corresponding author on reasonable request.

### 2.2 Sampling

A total of 22 patients (11 AS patients and 11 age‐matched non‐AS patients) were enrolled between April 2024 and July 2025 for sampling. Clinical trial number: not applicable. The surgical indications for AS patients included severe thoracolumbar kyphosis with a Cobb angle more than 50°, leading to restricted horizontal gaze, obvious cosmetic and daily functional impairment. All the non‐AS patients presented with spinal fracture and secondary neurological deficit requiring posterior decompression surgery. The uncalcified ligaments excised intra‐operatively were cleaned with sterile phosphate buffered saline (PBS) (Servicebio, China) for further processing.

### 2.3 Cell Isolation and Culture

After the removal of fat, muscle and connective tissue, the ligament tissue was washed with PBS and split into small pieces (less than a centimetre in diameter) with ophthalmic scissors. Then, the separated tissues were directly digested with 0.1% Type I collagenase (Gibco, California, USA) in the 37°C constant temperature shaker for 6–8 h till the tissue block was invisible. Thereafter, the digested solution was filtered through a 70 μm cell strainer (Falcon, China) to remove undigested tissue debris and the filtrate was collected into a centrifuge tube. After centrifuge at 1500 rpm for 5 min to the filtrate, resuspend the deposit in a fresh complete medium (DMEM [Gibco, USA] with 10% foetal bovine serum [Cellmax, China] and 1% penicillin and streptomycin [Gibco, USA]) and incubate it in a 75 cm^2^ cell culture flask (Corning, New York, USA) at 37°C and 5% CO_2_. Fresh medium was replaced after observing cell adherence within 24–48 h. Thereafter, the medium was changed every 2–3 days. Subculture was performed when cell density reached 80%–90%. Cells within 10 passages were selected for the experiments. Fbs at different passages were assigned to distinct experiments. In detail, **Passage 1–2 (P1‐P2)** Fbs were used for RNA‐seq, given that cells at these early passages preserved phenotypes closely resembling those in the original tissue; **Passage 3–5 (P3–P5)** Fbs with preserved strong differentiation capacity were used for osteogenic characterisation assays, including qPCR, Western blot, ALP staining, ARS and immunofluorescence; For biological replicates used in quantitative statistical analysis of Western blot and immunofluorescence, we included cells from **Passage 6–10 (P6–P10)**. Although gradual de‐differentiation and reduced expression of mature markers accompanied by progressive cellular senescence occurred with extended passaging up to P10, our validation data supported that the core phenotype of Fbs was still maintained across P1–P10, justifying their use for replicate quantification.

### 2.4 Osteogenic Differentiation Culture and Cell Treatments

When the cell density reached 80%–90%, the Fbs were seeded at a density of 2 × 10^5^ and 5 × 10^4^ cells per well in 6‐well and 24‐well plate, respectively. After culture for 24–48 h, the complete medium was switched to osteogenic differentiation medium consisting of complete medium, 0.1 μM dexamethasone (Solarbio, China), 50 μM ascorbic acid (Solarbio, China) and 10 mM b‐glycerophosphate (aladdin, China). The medium was changed every 2–3 days. To analyse the effect of inflammatory microenvironment, 0–100 ng/mL TNF‐α (Origene, China) was applied in the osteogenic medium. Osteogenic differentiation was assessed using alkaline phosphatase (ALP) (Beyotime, China) and alizarin red staining (ARS) (Cyagen, California, USA). Quantitative analysis of microimages for all the wells was performed using ImageJ (version 1.80).

To analyse the influences of Ly294002 (meilunbio, China) on the osteogenesis of AS‐Fbs, Ly294002 (50 μM) was added in the osteogenic medium 4 h before TNF‐α stimulation. To evaluate the effects of FAK inhibition on expression of osteogenic markers, 2.5, 5 and 10 μM of PF‐573228 (Ambeed, China) were added on AS‐Fbs. Actually, Ly294002 was a well‐established PI3K inhibitor, and it should not be described as a direct AKT inhibitor [24]. In detail, Ly294002 inhibited PI3K, leading to decreased PIP3 levels and subsequent reduction of AKT phosphorylation at Ser473. Theoretically, it exerted an indirect inhibitory effect. Therefore, in our study, we used LY294002 to suppress AKT phosphorylation indirectly via inhibition of PI3K, which was the standard and appropriate application of this compound.

### 2.5 Immunofluenrence Staining

Cells in 24‐well or tissue sections were fixed with 4% paraformaldehyde (PFA) (Servicebio, China) for 15 min and permeabilized with Triton X‐100 (Beyotime, China) for 20 min. After washing the cells/sections with PBS, they were blocked with 5% goat serum (Proteintech, China) for 1 h. Then, the cells/sections were incubated for 24 h at 4°C in primary antibodies against the following antigens: anti‐vimentin antibody (Abcam, UK; 1:200), anti‐FAP antibody (ABclonal, China; 1:200), anti‐α‐SMA antibody (ABclonal, China; 1:200), anti‐Runx2 antibody (Abcam, UK; 1:200), anti‐Col 1 antibody (ABclonal, China; 1:200), anti‐phospho‐AKT antibody (CST, USA; 1:400), anti‐phospho‐FAK antibody (Proteintech, China; 1:200), anti‐Thrombospondin 1 (THBS1) antibody (Abcam, UK; 1:200), anti‐Fibulin 1 (FBLN1) antibody (Proteintech, China; 1:100), anti‐OPN antibody (Proteintech, China; 1:200), anti‐OCN antibody (Bioss, China; 1:100). Thereafter, the cells/sections were washed three times with PBS and probed with secondary antibodies for 1 h at room temperature, including Alexa Fluor 488 goat anti‐rabbit (ABclonal, China; 1:200) and Alexa Fluor 594 goat anti‐mouse (ABclonal, China; 1:200). Optionally, the cytoskeleton staining was performed with TRITC labelled phalloidin (Yeasen, China; 1:200). Finally, following washing three times with PBS, the nuclear staining was implemented with DAPI (Servicebio, China). Cells positive for the target marker and relative mean fluorescence intensity in each field of view were counted using a fluorescence microscope (Zeiss, Germany). Quantitative analysis of the results was analysed with imageJ (version 1.80).

### 2.6 Immunohistochemistry

The paraffin‐embedded sections were deparaffinized in xylene (3 washes, 5 min each), followed by rehydration through a graded ethanol series (100%, 95%, 70%, 50% ethanol, 2 washes of 5 min per time) and rinsing in distilled water. Antigen retrieval was performed by immersing slides in 10 mM sodium citrate buffer (pH 6.0) (Servicebio, China) and microwaving for 15 min at near‐boiling temperature, then allowing them to cool to room temperature for 20 min. Following washing three times with PBS, the sections were incubated in 3% hydrogen peroxide (Beyotime, China) for 10 min to quench the endogenous peroxidase activity. Thereafter, the sections were blocked with 5% bovine serum albumin (BSA) in PBS for 30 min at room temperature to prevent non‐specific binding. Primary antibodies against TNF‐α (Servicebio, China; 1:200) diluted in blocking buffer were applied overnight at 4°C. After washing 3 times with PBST (10 min each), HRP‐conjugated secondary antibodies (Abcam, UK; 1:200) were incubated for 1 h at room temperature, followed by repeated washes. Freshly prepared brown diaminobenzidine (DAB) substrate solution (Servicebio, China) was used to visualize the protein localization with colour development monitored microscopically for 5 min before stopping with distilled water. Nuclear staining was performed with haematoxylin (Solarbio, China) for 10 s to 5 min, followed by dehydration via graded ethanol and xylene. The quantification was presented as bar graphs showing the percentage of positive cells or staining intensity scores. Besides, positive staining was defined as DAB signal clearly above background, with an intensity score ≥ 2 on a 0–3 scale (0 = negative, 1 = weak, 2 = moderate, 3 = strong). Only cells with moderate or strong staining were counted as positive. The threshold was established before blind quantification by two independent observers.

### 2.7 Quantitative Real‐Time PCR (RT‐qPCR)

Total RNA was isolated from the Fbs using TRIzol reagent (Invitrogen, USA) and transcribed into cDNA with a Evo M‐MLV RT Reaction Mix (AG, China) following the manufacturers' protocols. Quantitative real‐time PCR was conducted on a LightCycler480 PCR System (Roche, Switzerland) using a SYBR Green Premix Pro Taq HS qPCR Kit (AG, China). The relative expression levels of each gene were analysed by the 2^−△△Ct^ method. The primer sequences included: homo ALPL‐F, TGCATAACATCAGGGACATTGAC; homo ALPL‐R, TGGCTTTCTCGTCACTCTCATAC; RUNX2‐F, CACTGGCGCTGCAACAAGA; RUNX2‐R, CATTCCGGAGCTCAGCAGAATAA; OCN‐F, CCTCACACTCCTCGCCCTAT; OCN‐R, CGCCTGGGTCTCTTCACTAC; COL1A1‐F, TAGGGTCTAGACATGTTCAGCTTTG; COL1A1‐R, CGTTCTGTACGCAGGTGATTG.

### 2.8 RNA‐Sequencing

The RNA‐seq experiment was rigorously conducted following standard molecular biology protocols. Primary AS‐Fbs derived from ligament tissues were selected as the experimental material. The cells were divided into two groups: one group received no treatment (control), while the other was subjected to osteogenic induction for 72 h. After treatment, the cells were washed with ice‐cold phosphate‐buffered saline (PBS), and an appropriate volume of TRIzol reagent was added. The cells were thoroughly lysed by repeated pipetting until the cell‐lysis mixture became clear, non‐viscous, and free of cell clumps. Total RNA was then extracted. The concentration and purity of the extracted total RNA were precisely measured using a NanoDrop ND‐1000 spectrophotometer, with particular attention to the A260/A280 ratio, which was required to be between 1.8 and 2.0 to verify RNA purity. RNA integrity was further assessed using an Agilent 2100 Bioanalyzer, and the RNA integrity number (RIN) was calculated. All samples included in the analysis had RIN values greater than 7.0, indicating good integrity of the extracted total RNA and meeting the requirements for subsequent mRNA isolation and sequencing.

Raw sequencing reads underwent quality control to filter out low‐quality sequences and adapter contamination. High‐quality clean reads were then aligned to the reference genome. Based on the alignment results, gene expression levels were quantified and normalised as fragments per kilobase of transcript per million mapped reads (FPKM). Differentially expressed genes (DEGs) were identified using the following criteria: |log_2_(fold change)| > 0.5 and *p*‐value < 0.05. Heatmaps of DEGs, Gene Ontology (GO) and Kyoto Encyclopedia of Genes and Genomes (KEGG) pathway enrichment analyses, as well as Sankey diagrams, were generated using the clusterProfiler package in R. The heatmap was constructed based on the FPKM values of the two groups to visually represent the expression differences of DEGs across samples.

### 2.9 ShRNA Knockdown of Integrin Subunit Alpha 5 (ITGA5)

Adherent cells were seeded in 6‐well plates at a density of 1 × 10^5^ cells per well 24 h prior to transfection, ensuring 30%–50% confluence at the time of viral infection. Lentiviral particles encoding target genes (JikaiGene, China) were thawed on ice and appropriate volumes were added to each well to reach a multiplicity of infection (MOI) of 20. After gentle mixing, cells were incubated with the viral mixture in a 37°C and 5% CO_2_ incubator for 24 h. The medium containing lentivirus was then aspirated, and fresh complete medium was added to each well to avoid potential viral toxicity. After 48 h post‐transfection, efficiency was evaluated by observing fluorescence expression with the fluorescence microscope (Zeiss, Germany). 2 μg/mL puromycin (Beyotime, China) was added to the medium 72 h post‐transfection to select the stably transfected cells. The medium was changed every 2–3 days till all the untransfected cells were eliminated.

### 2.10 Western Blot

Total proteins were extracted from Fbs using cold RIPA lysis buffer (Servicebio, China) supplemented with protease and phosphatase inhibitors (Fudebio, China). The lysates were incubated on ice for 30 min and then centrifuged at 12,000 rpm for 15 min at 4°C to collect the supernatants. Protein concentrations were determined using the BCA assay (Beyotime, China), with absorbance measured at 562 nm. For sample preparation, 5 × loading buffer (Fudebio, China) was added, followed by denaturation at 95°C–100°C for 5 min.

SDS‐PAGE was performed using 10%–15% polyacrylamide gels (Vazyme, China). Electrophoresis was conducted at 80 V for 30 min during the stacking phase and 120 V for approximately 1 h during the separating phase. Proteins were then transferred onto methanol‐activated polyvinylidene difluoride (PVDF) membranes (Millipore, USA) using a wet transfer system at 320 mA for 1.5–2 h under ice‐cooled conditions.

After transfer, membranes were blocked with 5% non‐fat skim milk (or BSA for detecting phosphorylated proteins) in Tris‐buffered saline with Tween‐20 (TBST) for 1 h at room temperature. They were then incubated with primary antibodies specific to the target proteins at 4°C overnight, followed by three 15‐min washes with TBST. The primary antibodies against the antigens were listed as follows: anti‐Col 1 antibody (CST, USA; 1:1000), anti‐Runx2 antibody (CST, USA; 1:1000), anti‐OPN antibody (ABclonal, China; 1:1000), anti‐OCN antibody (ABclonal, China; 1:1000), anti‐FAP antibody (ABclonal, China; 1:1000), anti‐phospho‐AKT antibody (CST, USA; 1:2000), anti‐AKT antibody (Proteintech, China; 1:2000), anti‐THBS1 antibody (ABclonal, China; 1:1000), anti‐FBLN1 antibody (Proteintech, China; 1:1000), anti‐ITGA5 antibody (Abcam, UK; 1:1000), anti‐FAK antibody (Proteintech, China; 1:5000), anti‐phospho‐FAK antibody (CST, USA; 1:1000).

Corresponding HRP‐conjugated secondary antibodies were added and incubated for 1 h at room temperature, after which unbound antibodies were removed by repeated washes with TBST. GAPDH (ABclonal, China; 1:1000) was used as an internal control, ensuring that the molecular weight differed from that of the target proteins by at least 5 kDa, which was critical to avoid overlapping bands or signals. Protein bands were visualised using an ECL chemiluminescence detection kit (Abbkine, China) with an imaging system. Band intensities were quantified using ImageJ (version 1.80).

### 2.11 Animal Model

The digested fibroblasts were resuspended in an appropriate volume of osteogenic induction medium, followed by the addition of an equal volume of Matrigel (Corning, USA). After thorough mixing, the mixture was subcutaneously injected into 4‐week‐old nude mice. The mice were returned to their cages for continued rearing only after the subcutaneous transplants had solidified and formed. To maintain the local microenvironment, osteogenic induction medium treated with the cytokines or inhibitors was injected around the grafts every 2–3 days. Samples were harvested at 1 month, 2 months, and 3 months, respectively. All experimental procedures performed on mice were strictly in compliance with the regulations and guidelines governing the ethical use and care of animals. In terms of the protocol (peri‐graft injection of osteogenic induction medium containing cytokines or inhibitors every 2–3 days), actually, this was not a first attempt in our hands. The described protocol has already been practiced and reported by our prior publications for chondrocytes and BMSCs [25, 26]. In detail, via in vivo subcutaneous injection and transplantation, the formation of cartilage tissue or osteogenic potential‐possessed harvest tissue could be accomplished. In spite of this, we acknowledged that further validation and optimization of this protocol might be needed to provide a more acceptable alternative research model for HO.

In addition, several measures taken to prevent subcutaneous leakage of the cell‐Matrigel mixture and to ensure the reliable establishment of the ectopic ossification model should be emphasised: (1) First, the cell‐Matrigel mixture was prepared and maintained on ice to keep the Matrigel in a liquid state and prevent premature polymerisation. Immediately after implantation, the mixture was briefly maintained shaping with the use of spherical moulds or EP tubes; (2) Second, to prevent retrograde leakage through the needle track, the mixture was injected through a single puncture with multi‐directional deposition towards a common central point, thereby creating a consolidated subcutaneous depot. After withdrawal of the needle, the skin was gently smoothed, and the puncture site was slightly displaced relative to the underlying subcutaneous tissue to seal the needle track (a principle analogous to the Z‐track injection technique), which effectively prevents the injectate from overflowing through the puncture channel.

### 2.12 μ‐CT Analysis

A Micro‐CT scanner (mCT80; Scanco Medical AG, Switzerland) was utilised to analyse the harvested tissue from nude mice. Two‐dimensional (2D) and three‐dimensional (3D) images were reconstructed using Scanco Medical software.

### 2.13 Histological Staining

Haematoxylin and eosin (HE), Masson, Sirius Red, Safranin O/fast green (SOFG) or Methylene blue acid fuchsin (MB‐AF) (Solarbio, China) for sections from spinal ligaments or harvested transplanted tissues were performed following the instructions. In terms of MB‐AF staining, it was mainly used for the morphological observation of bone tissues, particularly undecalcified bone sections. In detail, stain dark blue or dark purple with distinct outlines staining indicated cell nuclei of osteoblasts. Besides, deep red areas represented newly formed mineralised bone, whereas purplish grey areas referred to incompletely mineralised osteoid secreted by cells.

### 2.14 Cell Viability Analysis

For assessing cell viability using the cell counting Kkit‐8 (CCK‐8) assay, exponentially growing cells were first harvested and adjusted to a suitable density. Typically, 1 × 10^3^ cells in 100 μL complete medium were seeded per well in 96‐well plates, with peripheral wells filled with PBS to minimise edge effects. For the experimental group, various concentrations of TNF‐α, Ly294002 or PF‐573228 were added in the medium. Following treatment incubation (based on experimental design), 10 μL of CCK‐8 reagent (Meilunbio, China) was added to each well against exposure to light conditions to prevent Water‐Soluble Tetrazolium Salt‐8 (WST‐8) degradation. The plate was gently returned to the incubator for 1–2 h at 37°C with 5% CO_2_ and then the absorbance measurements at 450 nm were measured. Cell viability was calculated using the formula: [(Experimental OD—Blank OD)/(Control OD—Blank OD)] × 100%.

### 2.15 Statistical Analysis

All experimental data were obtained from duplicate or triplicate experiments with at least three independent repetitions, and are presented as mean ± standard deviation (SD). For quantitative analyses, a minimum of three biological replicates were included. In this study, ‘*n*’ represented the number of biological replicates and was defined as independently established primary Fb cultures derived from individual patients (evaluated as independent experimental units). Technical replicates were averaged within each biological replicate and were not included in the sample size. To specify, although 22 patients (11 AS and 11 controls) were enrolled overall, the primary cell lines with relatively stable growth, morphology, and phenotype were finally selected for the experiments. These cells were expanded independently to sufficient cell amount to cover the subsequent experiments. Differences between two groups were analysed using a two‐tailed unpaired Student's *t*‐test. For multiple comparisons, one‐way analysis of variance (ANOVA) was applied: first, Levene's test was used to assess homogeneity of variance, followed by Bonferroni post hoc test as appropriate based on the specific comparisons and statistical requirements. A *p*‐value < 0.05 was considered statistically significant. All data were summarised and analysed using GraphPad Prism 10 (GraphPad Software, USA) and SPSS software (version 12.0, 2003; SPSS Inc., Chicago, IL, USA).

## 3 Results

### 3.1 Isolation and Identification of Fbs Derived From Uncalcified Spinal Ligaments

To investigate the underlying mechanisms of pathological ectopic ossification in the spinal ligaments, uncalcified ligament tissues were collected from age‐ and sex‐matched AS and thoracolumbar fracture patients, who underwent 3‐column osteotomy and posterior decompression surgery, respectively (Figure 1A). Haematoxylin–eosin (H&E), Masson, Safranin O/Fast Green (SOFG), and Sirius Red staining were used to reveal the structural differences of ligaments between AS and control group. The spinal ligaments from AS patients exhibited more pronounced signs of osteogenic differentiation (the black triangles) and a greater amount of disorganised collagen fibre than that of the fracture group (the black arrows) (Figure 1B).

**FIGURE 1:**
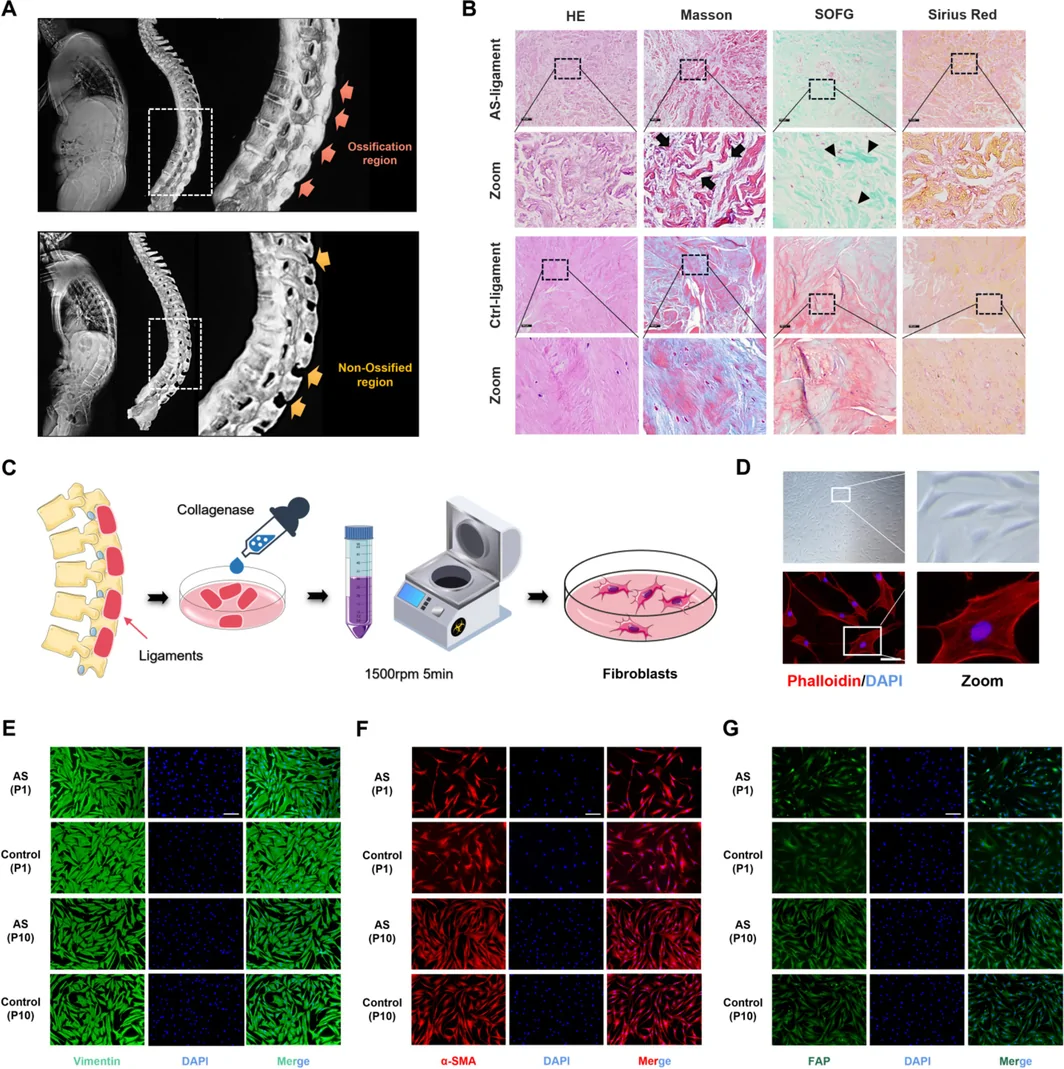
Isolation and identification of Fbs derived from the unossified spinal ligaments. (A) Radiographs and CT reconstruction showed the ossified and non‐ossified region in spinal ligament of AS. The yellow arrows indicated the site for sampling. (B) H&E, Masson, SOFG and Sirius Red staining of the AS spinal ligaments showed superior osteogenic potential and more disorganised collagen arrangement structure than that of the control group. Scale bar: 100 μm. (C) Illustration of the isolation process for the spinal ligament‐derived Fbs. (D) Morphology of Fbs under bright field and cytoskeleton staining. Scale bar: 50 μm. (E–G) Identification of AS‐Fbs and control‐Fbs (cells at passage 1 and 10) using representative biomarkers via immunofluorescence staining images, including Vimentin, α‐SMA and FAP. Scale bar: 100 μm. As, ankylosing spondylitis; FAP, fibroblast activation protein; Fbs, fibroblasts; H&E, haematoxylin–eosin; SOFG, Safranin O‐Fast Green; α‐SMA, alpha smooth muscle Actin.

Cytoskeletal staining was applied to show the cellular morphology of the cells isolated from non‐ossified spinal ligaments. Microscopic observation revealed that these cells manifested as a spindle‐shaped or polygonal morphology, combined with slender protrusions on their surface interconnecting with adjacent cells and forming a network‐like structure (Figure 1C,D). Such features were consistent with the basic structural characteristics of Fbs. Besides, immunofluorescence (IF) analyses were used to further determine the purity of cells via three typical biomarkers involving vimentin, alpha smooth muscle actin (α‐SMA) and fibroblast activation protein (FAP), showing a nearly 100% positive rate (Figure 1E–G). Furthermore, considering that repeated subculture might lead to phenotypic loss and drift in cells, accompanied by weakened differentiation capacity and abnormal differentiation direction, especially for cells at passages higher than 10, we compared the expression of aforementioned Fbs‐specific markers in cells at passage 1 and 10 via IF staining. The results clearly showed that at both passages, the proportion of positive cells remained nearly 100%, with no detectable loss of the phenotype (Figure 1E–G). All these results indicated that following the cell culture, passage, and purification, the predominant cell population isolated from the non‐ossified ligaments were fibroblasts, which served as the basis for subsequent experiments.

### 3.2 AS‐Fbs Exhibited Aberrant Osteogenic Transdifferentiation Potential and Self‐Osteogenic Capacity

In order to confirm the abnormal osteogenic transdifferentiation potential of AS‐derived Fbs, ALP and ARS staining were used to elucidate the osteogenic differentiation signs visually. The results showed a superior osteogenic capacity of AS‐Fbs than that of the control group (Figure 2A,B), indicating the critical role of Fbs in the mechanism of pathological osteogenesis and ossification. RT‐qPCR and western blot analysis showed relatively higher expression levels of representative osteogenic mRNA and proteins (Runx2/Col 1/OPN/OCN) in AS‐derived Fbs than that of the control cells during the process of osteogenic differentiation (Figures 2C–F and S1A). Moreover, instead of the relative intensity of IF images, the nuclear translocation rate of Runx2 was defined as the percentage of Fbs in which Runx2 IF signals predominantly localised to the nucleus (Runx2 signals combined with DAPI‐labelled nuclei), relative to the total number of cells counted. The IF images showed a greater rate of nuclear co‐localization and translocation of Runx2 in the AS group than in the normal group 3 days after osteogenic stimulation (OS) (Figure 2G). The CCK‐8 assay was performed to evaluate the proliferation rate. The AS‐Fbs manifested an enhanced proliferative capacity with or without osteogenic induction (Figure 2H). Notably, significant increased expression levels of FAP were found after OS in immunofluorescence and western blot images, which were regarded as one of the activation markers of Fbs, indicating its involvement in the aberrant osteogenesis of AS (Figures 2I,J, and S1B,C). Also, FAP was barely detectable in most normal adult tissues but was selectively up‐regulated in reactive Fbs during tissue remodelling, fibrosis, wound healing, and chronic inflammation. Considering the pathological new bone formation in AS arises at entheses through an inflammatory‐fibro‐osteogenic sequence in which activated Fbs were key cellular drivers, we reasoned that monitoring FAP expression would allow us to determine whether AS‐Fbs acquire an activated, ECM‐remodelling phenotype during osteogenic differentiation.

**FIGURE 2:**
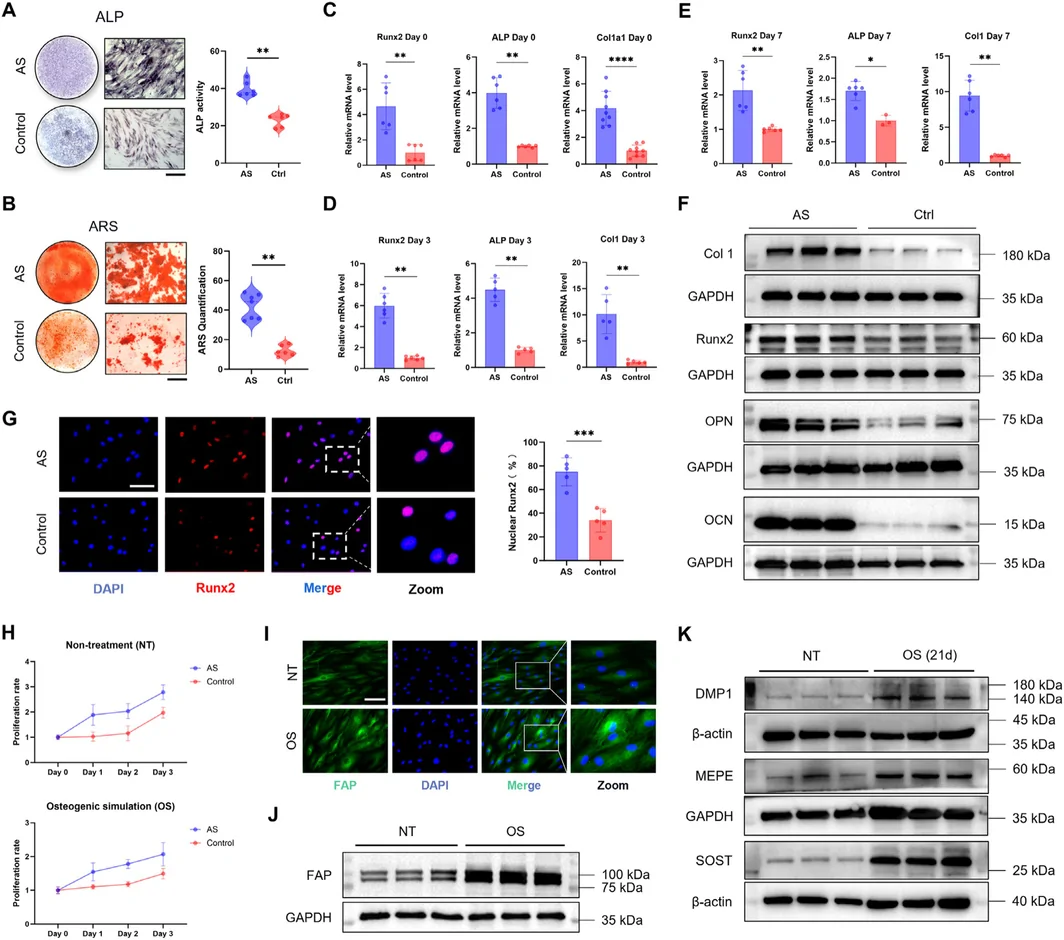
Superior osteogenic transdifferentiation potential and spontaneous osteogenic ability of AS‐derived Fbs than the control group. (A) ALP staining and quantitative analysis of Fbs from AS and control patients after 7 days of osteogenic stimulation. *n* = 6 per group. Scale bar: 300 μm. (B) ARS staining and quantitative analysis of Fbs from AS and control patients after 21 days of osteogenic stimulation. *n* = 6 to 7 per group. Scale bar: 300 μm. (C–E) Relative mRNA expression levels of osteogenic markers (Runx2, ALP and Col1a1) in Fbs from AS and fracture patients at 0, 3 and 7 days after osteogenic induction. (F) Difference of expression levels in Col 1, Runx2, OPN and OCN between Fbs from AS and control groups were analysed using western blotting. (G) Immunofluorescence staining and quantitative evaluation showed the nuclear co‐localization and translocation rate of Runx2 for AS and control Fbs following 3‐day osteogenic stimulation. *n* = 5 per group. Scale bar: 300 μm. (H) CCK‐8 assay of proliferative capacity for AS and control Fbs in non‐treatment and osteogenic stimulation conditions. (I, J) Representative immunofluorescence images and western blots of protein levels of FAP for AS‐Fbs before and after osteogenic induction. (K) Western blots of osteocytes‐derived secreted proteins (DMP1, MEPE and SOST) in AS group at day 0 (D0) and day 21 (D21) following osteogenic induction. Scale bar: 300 μm. ****p < 0.0001, ***p < 0.001, **p < 0.01, *p < 0.05. ALP, alkaline phosphatase; ARS, alizarin red; AS, ankylosing spondylitis; DMP1, dentine matrix acidic phosphoprotein 1; FAP, fibroblast activation protein; Fbs, fibroblasts; MEPE, matrix extracellular phosphoglycoprotein; NT, Non‐treatment; OCN, osteocalcin; OPN, osteopontin; Runx2, runt‐related transcription factor 2; SOST, sclerostin.

In addition, in spite of the ARS demonstrating the osteoblast maturation and matrix mineralisation, the further differentiation of osteoblasts into mature osteocytes should be investigated [27, 28, 29]. We assessed the expression of osteocyte‐specific secreted proteins (DMP1, dentine matrix acidic phosphoprotein 1; MEPE, matrix extracellular phosphoglycoprotein; SOST, sclerostin) in AS group at day 0 (D0) and day 21 (D21) following OS. Western blot analysis showed remarkable up‐regulation of all three target proteins at D21 versus D0, confirming successful trans‐differentiation of Fbs towards the osteocyte lineage (Figures 2K and S1D). These results suggested that AS ligaments‐derived Fbs possessed greater osteogenic capacity than that of the normal populations.

### 3.3 Relative Low Concentrations of TNF‐α Sustainably Enhanced the Osteogenic Activity of AS Fbs

To determine the influence of inflammatory cytokines on the osteogenic activity of AS Fbs, various concentrations of TNF‐α (0–100 ng/mL) were used for osteogenic stimulation, with the highest osteogenesis potential seen at the relatively low‐dose TNF‐α of 10 ng/mL. Results of ARS and ALP staining showed that the relatively low concentration of TNF‐α could persistently enhance the osteogenic capacity of AS Fbs, while the osteogenic characterisation was suppressed at high TNF‐α concentrations of 50 ng/mL and 100 ng/mL (Figure 3A–C). In contrast, Fbs from the control group exhibited no differences in osteogenic phenotypes (ALP and ARS) under graded inflammatory cytokine stimulation, revealing the specific response to inflammatory microenvironments and sensitivity to osteogenic induction of AS‐Fbs, combined with its involvement in the AS‐associated osteogenic transdifferentiation process (Figure S2A,B). Meanwhile, TNF‐α promoted the rate of Fbs proliferation at low concentrations (10 ng/mL) (Figure 3D). The western blotting and RT‐qPCR analysis showed that the relatively low concentrations of TNF‐α (10 ng/mL) could also promote the expression levels of osteogenic RNA and proteins, while higher doses of TNF‐α significantly suppressed the levels of the aforementioned markers (Figures 3E,F and S2C). Besides, IF images revealed a relatively higher nuclear co‐localization and translocation of Runx2 rate together with Col 1 expression level under low‐TNF‐α concentration stimulation than that of the higher inflammatory cytokine intervention group (Figure 3G–I). These findings indicate that the optimal TNF‐α concentration was 10 ng/mL to provide an appropriate pro‐inflammatory environment for the promotion of AS Fbs osteogenesis. All the subsequent experiments would be conducted on the basis of low dose TNF‐α stimulation. In addition, higher expression levels of TNF‐α in uncalcified AS spinal ligaments than in the control group indicated residual scattered inflammatory infiltration within ligament tissues from AS patients, which might mediate the progression of ossification and align with the in vitro observation that low concentrations of TNF‐α promoted pathological osteogenesis (Figures 3J and S2D). Collectively, these results indicated that under a relatively low‐concentration inflammatory microenvironment, the osteogenic differentiation of AS‐Fbs could be significantly enhanced.

**FIGURE 3:**
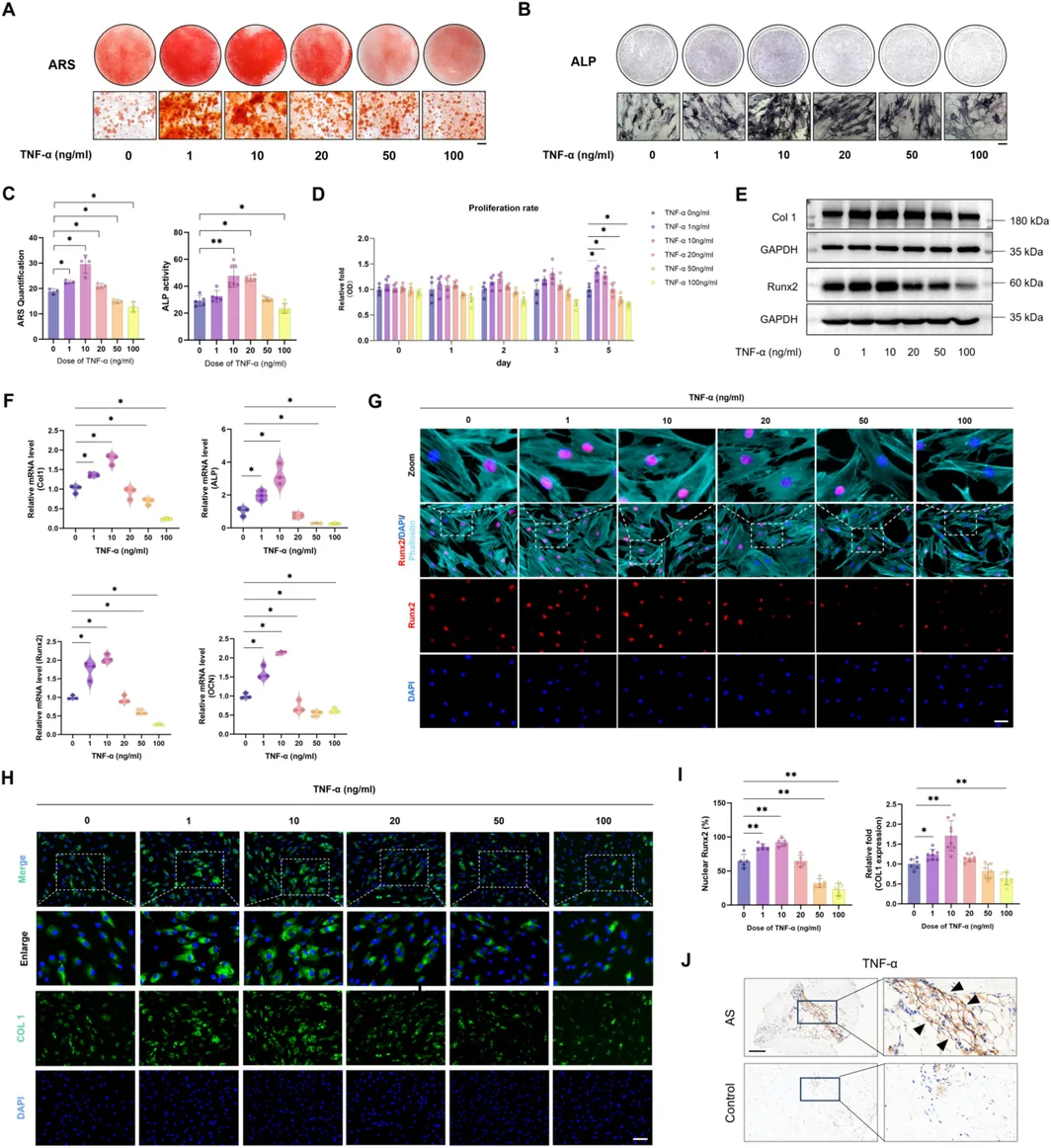
Effects of various‐concentration TNF‐α on osteogenic differentiation of AS‐derived Fbs. (A) ARS staining of pathologic ossification for AS‐Fbs with various dose of TNF‐α stimulation after 21‐day OS. Scale bar: 100 μm. (B) ALP staining of pathologic osteogenesis for AS‐Fbs with different dose of TNF‐α stimulation after 7‐day OS. Scale bar: 100 μm. (C) Quantitative analysis of ARS and ALP staining. (D) A CCK‐8 assay used for cell proliferation rate analysis with TNF‐α stimulation from 0 to 100 ng/mL. (E) Western blots of expression levels of typical osteogenic markers (Runx2/Col1) after OS with low‐to‐high concentration of TNF‐α treatment. (F) RT‐qPCR analysis of the mRNA levels for Col1A1, Runx2, ALP and OCN at 7 days of OS. (G,H) Immunofluorecence images of Runx2 (red) and Col 1 (green) in AS‐Fbs with 0–100 ng/mL TNF‐α stimulation for 72 h of OS. Scale bar: 100 μm. (I) Mean fluorescence intensity of Col 1, nuclear co‐localization and translocation rate of Runx2 in arbitrary units of (G, H). (J) Immunochemistry staining of TNF‐α for AS and control spinal ligaments. Scale bar: 300 μm. **p < 0.01, *p < 0.05. ALP, alkaline phosphatase; ARS, alizarin red; AS, ankylosing spondylitis; CCK‐8, cell counting Kkit‐8; Col 1, collagen I; Fbs, fibroblasts; OCN, osteocalcin; OPN, osteopontin; OS, osteogenic stimulation; RT‐qPCR, real‐time reverse transcription‐PCR; Runx2, runt‐related transcription factor 2; TNF‐α, tumour necrosis factor‐alpha.

### 3.4 AS‐Fbs Promoted Pathological Osteogenesis by Activating PI3K/AKT Signalling Pathway

To explore the downstream pathways associated with pathological osteogenic differentiation of AS‐Fbs, the marker of PI3K‐AKT signalling pathway was detected. Notably, our results showed a trend of increasing protein levels of phospho‐AKT after the osteogenic induction in AS‐Fbs (Figures 4A and S3A). In the present study, phosphorylated AKT (p‐AKT) expression was quantified across an acute time window from baseline (0 min) to 4 h following osteogenic stimulation. The selection of this minute‐scale detection time‐frame was justified on the following aspects [30, 31, 32]: (1) First, in line with the established literature, stimulus‐evoked protein phosphorylation represented a rapid, post‐translationally regulated event that proceeded on a minute‐to‐hour timescale, rather than a sustained, steady‐state alteration in total protein abundance. For instance, Li et al. demonstrated that PLCγ phosphorylation was markedly upregulated as early as 15 min after osteogenic induction and remained stable across multiple time points within a 2‐h observation period. (2) Second, as a proximal upstream transducer in the osteogenic signalling cascade investigated herein, AKT phosphorylation constituted one of the earliest intracellular signalling events triggered by osteogenic cues. Its rapid and sustained activation served as a critical prerequisite for initiating downstream osteogenic transcription. (3) Third, the time‐course data (0–4 h) in the current study further revealed that this signalling pathway was persistently activated during the early phase of osteogenesis and played a dominant regulatory role in driving the progression of osteogenic differentiation. In contrast, the osteogenic phenotypes characterised in preceding assays (ALP activity and ARS) reflected late‐stage functional readouts that manifested only after transcription. Accordingly, we specifically measured p‐AKT levels at 30–240 min post‐stimulation to capture the immediate upstream signalling cascade elicited by our experimental manipulations. Meanwhile, in comparison to 0 and 100 ng/mL concentration of TNF‐α stimulation, the AS‐Fbs treated with OS and 10 ng/mL showed greater expression levels of phospho‐AKT in immunofluorescence images 30 min post‐induction (Figure 4B).

**FIGURE 4:**
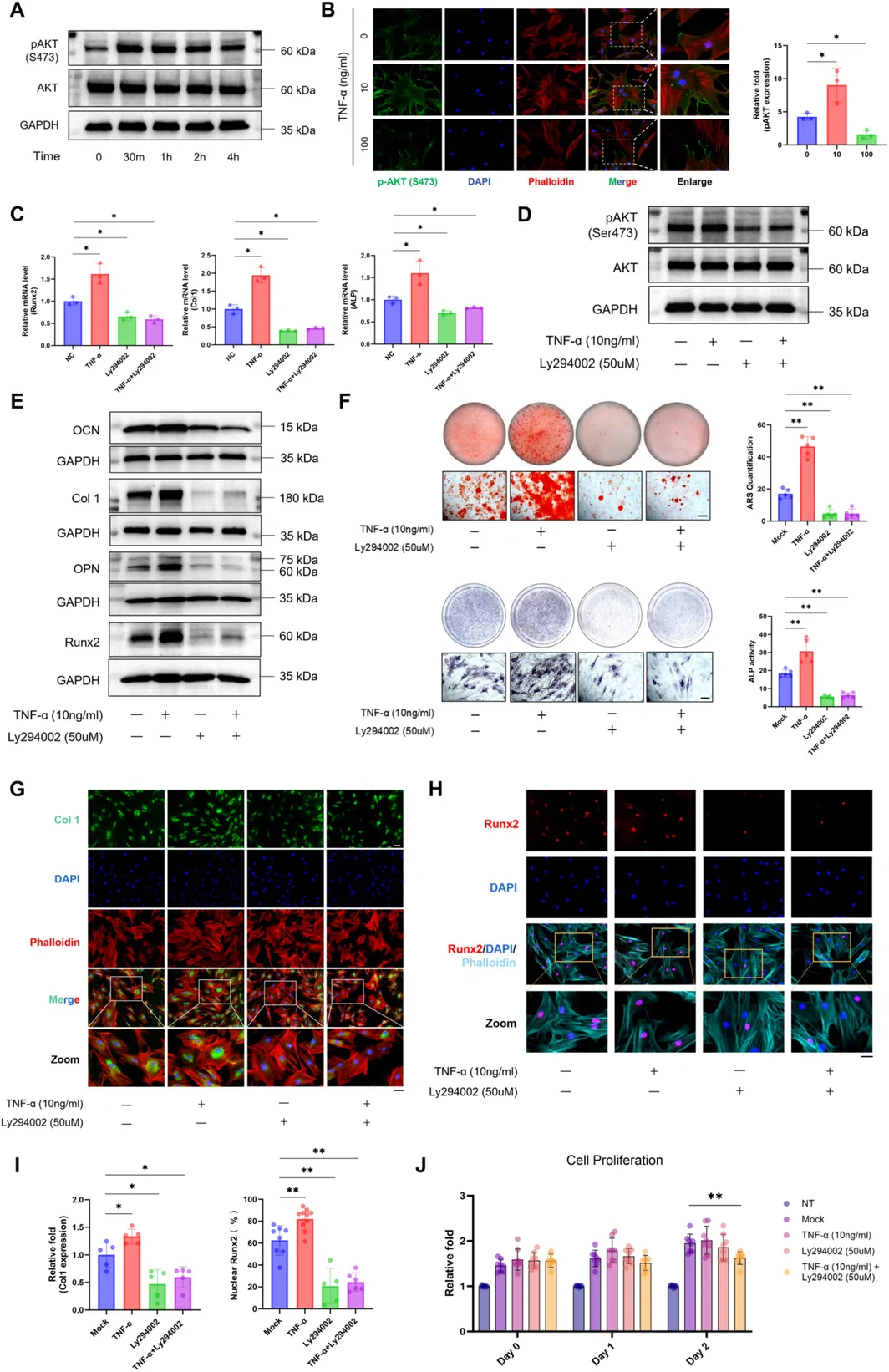
AS‐Fbs promoted osteogenic differentiation by activating PI3K‐AKT signalling. (A) Western blot analysis of expression levels for phospho‐AKT (Ser473) during the osteogenic induction. (B) Immunofluenrence imaging and quantitative analysis showed higher expression level of phospho‐AKT (Ser473) in low TNF‐α concentration group (10 ng/mL) than that of the other two groups with inflammatory stimulation of 0 and 100 ng/mL. Scale bar: 100 μm. (C) The mRNA expression levels of Runx2, Col1 and ALP in AS‐Fb with or without TNF‐α/Ly294002 stimulation. (D, E) Western blots evaluation of the expression levels for phospho‐AKT (Ser473), OCN, OPN, Col1 and Runx2 in AS‐Fbs after TNF‐α and Ly294002 intervention. (F) ARS, ALP staining and relevant quantitative assessment of AS‐Fbs treated with TNF‐α and/or Ly294002. Scale bar: 200 μm. (G–I) Immunofluenrence staining and quantitative assessment of the expression levels of Col 1 together with the nuclear co‐localization and translocation of Runx2 rate, respectively. Scale bar: 100 μm. (J) The CCK assay showed the cell proliferation of AS‐Fbs with the intervention of TNF‐α and Ly294002 during the 2‐day osteogenic induction. **p < 0.01, *p < 0.05. ALP, alkaline phosphatase; ARS, alizarin red; AS, ankylosing spondylitis; CCK‐8, cell counting Kkit‐8; Col 1, collagen I; Fbs, fibroblasts; OCN, osteocalcin; OPN, osteopontin; OS, osteogenic stimulation; Runx2, runt‐related transcription factor 2.

Then, the specific inhibitor for PI3K/AKT pathway (Ly294002, 50 μM) was applied to further validate the signalling activation during the osteogenesis of AS‐Fbs. The administration of Ly294002 could significantly decrease the mRNA levels of Runx2, Col1 and ALP 3 days post‐induction (Figure 4C). Western blotting analysis also revealed the inhibition of expression levels for phospho‐AKT (30 min post‐induction) and representative osteogenesis‐associated markers (Runx2/Col 1/OPN/OCN) following the use of Ly294002 (Figures 4D,E and S3B,C). The time‐points for the evaluation of Runx2, Col1, OPN and OCN were 3, 3, 7 and 14 days after osteogenic stimulation. Assessment of ALP and ARS staining also demonstrated inhibited osteogenic potential induced by Ly294002 (even under circumstance of low‐dose TNF‐α stimulation), confirming the relationship between pathological osteogenesis of AS‐Fbs and abnormal activation of PI3K/AKT pathway (Figure 4F). Besides, the results of immunofluenrence staining revealed reduction of nuclear Runx2 translocation rate and expression level of Col 1 after 3‐day OS and Ly294002 intervention, verifying the predominant role of PI3K/AKT signalling in promotion of pathologic ossification (Figure 4G–I). In addition, the CCK‐8 assay showed a partially inhibited cell proliferation rate in AS‐Fbs with Ly294002 treatment but without obvious cytotoxic effects (Figure 4J).

Furthermore, due to the role of PI3K as an upstream of AKT and PI3K regulated multiple other downstream effectors, the application of an upstream inhibitor (Ly294002) was not sufficiently rigorous to confirm the specific role of the downstream FAK/AKT axis. MK‐2206, a direct AKT inhibitor, was also adopted. According to the osteogenic phenotypes, MK‐2206 treatment markedly suppressed AKT phosphorylation (even stronger than the Ly294002), reduced ALP activity and mineralised nodule formation, and down‐regulated osteogenic marker expression during osteogenic differentiation of AS‐Fbs, which independently strengthened the causal role of the FAK/AKT axis rather than off‐target effects from upstream PI3K inhibition (Figure S3D–F). These results strongly suggested the essential role of the PI3K/AKT pathway in the process of pathological osteogenesis of AS‐Fbs.

### 3.5 Abnormal Osteogenic Differentiation of AS‐Fbs Depended on the Activation of ITGA5 Mediated by Secreted ECM

To determine the upstream signalling of the PI3K/AKT pathway and detailed molecular mechanisms of pathological osteogenic differentiation in AS, RNA sequencing for AS‐Fbs with and without osteogenic induction was performed. The differentially upregulated genes related to ECM including ITGA5, FBLN1, and THBS1 were selected for further study (Figure 5A). Go‐term analysis revealed enrichment of gene sets involving extracellular region/space, collagen‐containing extracellular matrix, and extracellular matrix structural constituent (Figure S4A). Regarding the selection of candidate DEGs, the underlying rationale was detailed as follows: (1) First of all, the filtering criteria (|log2 (fold change)| > 0.5 and *p*‐value < 0.05) were applied. Under this threshold, both THBS1 and FBLN1 were identified among the most significantly up‐regulated transcripts in the experimental conditions relative to controls. (2) Second, our study centered on the fibrous‐to‐osseous tissue transition during HO. THBS1 and FBLN1, as ECM glycoproteins, were involved in the ECM organisation, cell‐matrix interactions, and collagen deposition. Their biological roles were highly consistent with the core mechanism investigated in this work. (3) Most importantly, a panel of ECM proteins (e.g., Col11a1, SVEP1, PTX3) was initially selected and tested in our pre‐experiments. Following the repeated validation and preliminary findings, merely THBS1 and FBLN1 were confirmed to significantly enhance the osteogenic capacity of AS‐Fbs. Taken together, the final selection of candidate genes was determined by their expression abundance, functional relevance, and experimental attempts. KEGG assessment identified pathway enrichment including cytoskeleton in muscle cells, PI3K‐Akt signalling pathway, and ECM‐receptor interaction (Figure S4B). RNA sequencing mulberry plot for representative upregulated genes showed the enrichment in the PI3K‐AKT signalling pathway, which was consistent with (Figures 4 and 5B). Within the 0–7 day time of osteogenic induction, western blotting analysis revealed increasing expression levels of THBS1 and FBLN1 (Figures 5C and S4C). Moreover, a relatively higher level of THBS1 and FBLN1 was detected in AS‐Fbs than that of the control ones (Figures 5D and S4D). Immunofluenrence images also confirmed the increase of THBS1 and FBLN1 level after 2 days of OS (Figure 5E,F). Then, to validate the relationship between the aforementioned ECM proteins and osteogenesis, the AS‐Fbs were stimulated with THBS1 and FBLN1 proteins during the osteogenic induction. Results of ALP and ARS staining analysis showed enhanced osteogenic signs in AS‐Fbs with ECM stimulation (Figure 5G,H).

**FIGURE 5:**
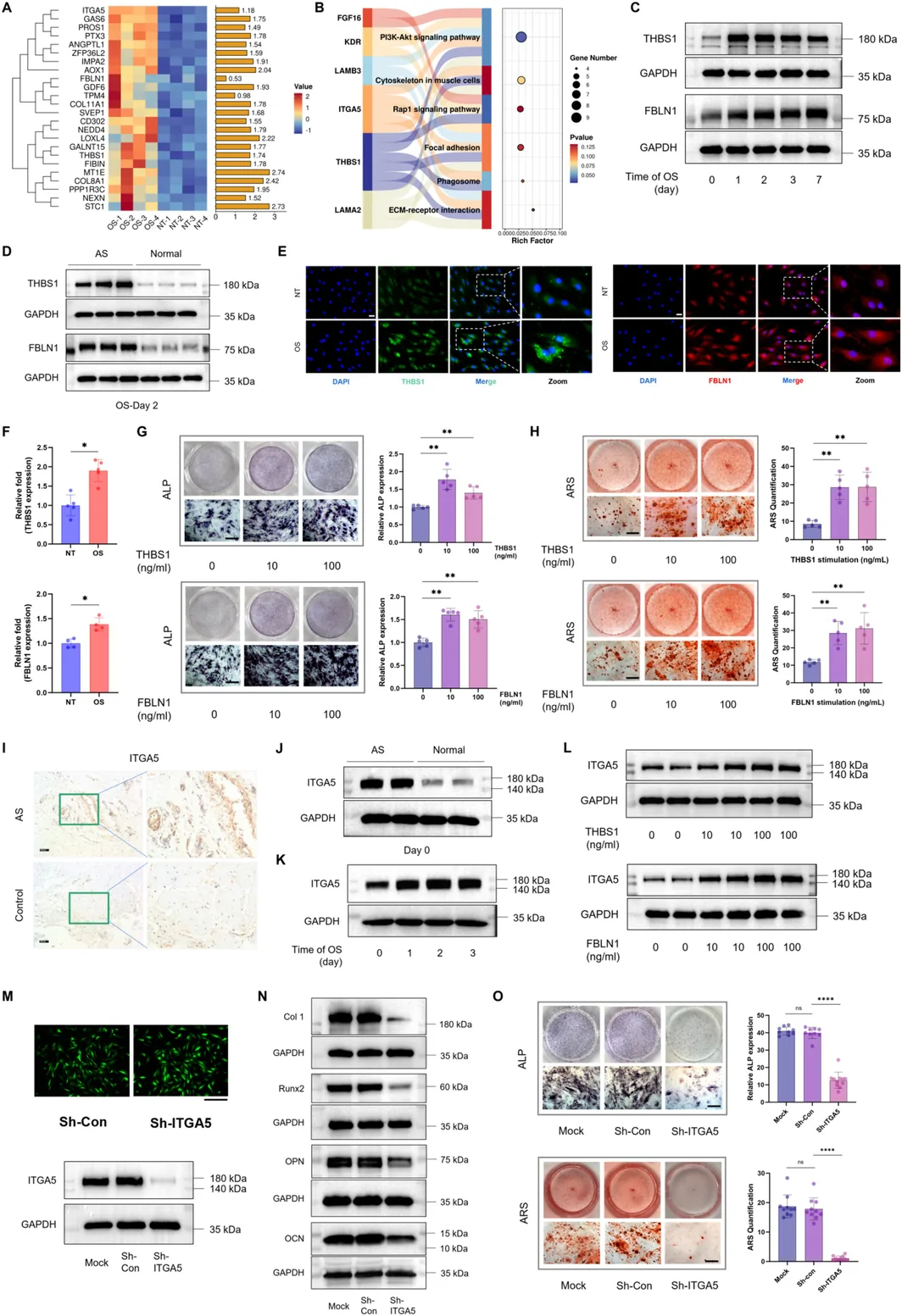
Abnormal osteogenic differentiation of AS‐Fbs depended on the activation of ITGA5 mediated by ECM. (A) Heatmap of differentially upregulated genes between cells treated with and without osteogenic stimulation. (B) RNA sequencing Mulberry plot of representative upregulated genes. (C) Western blots showed the OS time‐depended expression levels of THBS1 and FBLN1. (D) Comparison of expression levels for THBS1 and FBLN1 between AS and control‐Fbs at 2‐day osteogenic stimulation. (E, F) Immunofluenrence images and relevant quantification of THBS1 and FBLN1 levels before and after OS. Scale bar: 100 μm. (G, H) ALP, ARS staining and quantitative analysis for AS‐Fbs with THBS1 and FBLN1 stimulation (concentration ranged from 0 to 100 ng/mL) after 7‐ and 21‐day OS, respectively. Scale bar: 300 μm, *n* = 5 per group. (I) Immunohistochemical staining of ITGA5 in AS and control spinal ligament tissues. Scale bar: 100 μm, *n* = 4 per group. (J) Western blot evaluation for ITGA5 in AS and control‐Fbs. (K) Western blots of ITGA5 levels for AS‐Fbs with 0 to 3 days OS. (L) Western blots for expression levels of ITGA5 in AS‐Fbs with stimulation of THBS1 and FBLN1 (0–100 ng/mL) for 4 h. (M) Immunofluenrence for transfection efficiency and western blot for knock‐down efficiency in the AS‐Fbs treated with PBS, Sh‐control and Sh‐ITGA5 transfection. Scale bar: 300 μm. (N) Western blots of Col1, Runx2, OPN and OCN levels after Sh‐ITGA5 transfection. (O) ALP, ARS staining and relative quantification for AS‐Fbs with Sh‐ITGA5 transfection after 7‐ and 21‐day OS, respectively. Scale bar: 300 μm. ****p < 0.0001, **p < 0.01, *p < 0.05, ns: *p* > 0.05. ALP, alkaline phosphatase; ARS, alizarin red; AS, ankylosing spondylitis; Col 1, collagen I; FBLN1, Fibulin 1; Fbs, fibroblasts; ITGA5, Integrin alpha 5; OCN, osteocalcin; OPN, osteopontin; OS, osteogenic stimulation; Runx2, runt‐related transcription factor 2; THBS1: Thrombospondin 1.

To further verify the role of ITGA5 in the osteogenesis of AS‐Fbs, its expression levels for the difference between AS and control groups were detected. The results of western blotting and immunochemistry staining revealed a higher level of ITGA5 in AS‐Fbs and spinal ligaments, respectively (Figures 5I,J and S4E,F). Meanwhile, significant increasing level of ITGA5 was found after osteogenic induction (Figures 5K and S4G). Besides, administration of THBS1 and FBLN1 could promote the activation and expression of ITGA5 in a short period (4 h) (Figures 5L and S4H). Furthermore, to illustrate the correlation between ITGA5 activation and osteogenesis from another perspective, ShRNA of ITGA5 was transfected in AS‐Fbs. With the efficient transfection of Sh‐ITGA5, the expression of representative osteogenic markers were reduced 2 days post‐transfection (Figures 5M,N and S4I,J). ALP and ARS images also demonstrated obvious weakened osteogenic characterisation following the knock‐down of ITGA5 (Figure 5O).

In addition, considering that the RNA‐sequencing was performed to compare AS‐Fbs ‘with and without osteogenic induction’, without introducing the low‐dose TNF‐α variable. The additional validation was required to confirm that the ECM/ITGA5 axis identified by RNA‐seq was responsive to low‐dose TNF‐α stimulation. Therefore, the expressions of THBS1, FBLN1 and ITGA5 in AS‐Fbs under osteogenic induction with three treatment conditions: control, low‐dose TNF‐α (10 ng/mL), and high‐dose TNF‐α (100 ng/mL) were detected. In consistent with our hypothesis, the results demonstrated that low‐dose TNF‐α significantly upregulated THBS1, FBLN1 and ITGA5 protein levels, whereas high‐dose TNF‐α induced inhibition effects to expression of these proteins. These data directly verified that the ECM/ITGA5 axis found from RNA‐seq was specifically hyper‐activated under the low‐dose TNF‐α inflammatory microenvironment, bridging the ‘inflammatory microenvironment’ findings to transcriptomic signature (Figure S4K). All these findings emphasise the necessity of ECM production and associated ITGA5 activation in the osteogenic differentiation for AS‐Fbs.

### 3.6 AS‐Fbs Promoted Osteogenic Differentiation Through FAK‐Phosphorylation Medicated by ITGA5

Focal adhesion kinase (FAK), typically functioning as a signal transduction hub, could bridge extracellular microenvironmental signals and intracellular functional pathways to regulate key biological processes including cell migration, proliferation, and differentiation [33, 34, 35, 36]. Therefore, to further investigate the specific signals that mediate the PI3K/AKT pathway after activation of ITGA5 for AS‐Fbs, the level of FAK phosphorylation was measured. During the 0–4 h of osteogenic induction, the expression levels of phospho‐FAK (p‐FAK, Y397) exhibited a statistically significant upward trend, indicating the recruitment and activation of FAK after osteogenic induction (Figures 6A,B and S5A). To elucidate the underlying correlation between FAK and osteogenesis, the specific FAK inhibition—PF573228 was applied. ALP and ARS staining images showed significant concentration‐dependent reduction of osteogenic signs with use of PF‐573228 ranged from 0 to 10 μM (Figure 6C). Moreover, the reduced protein levels of phosphorylated AKT were found via western blotting assessment after FAK inhibition, verifying the role of PI3K/AKT as the downstream effector (Figures 6D and S5B). The decreased levels of representative osteogenesis‐related markers and a slight inhibiting function of PF‐573228 for cell proliferation of AS‐Fbs were also identified, consistent with aforementioned findings (Figures 6E–G and S5C). To further confirm the significance of ITGA5 activation to the downstream signal pathways, we conducted western blotting analysis experiments. We observed the decreased expression levels of p‐FAK and p‐AKT after the knockdown of ITGA5 with ShRNA transfection (Figures 6H and S5D). In conclusion, our results underscored the FAK‐phosphorylation and ITGA5/FAK/AKT signalling axis in the osteogenesis of AS‐Fbs.

**FIGURE 6:**
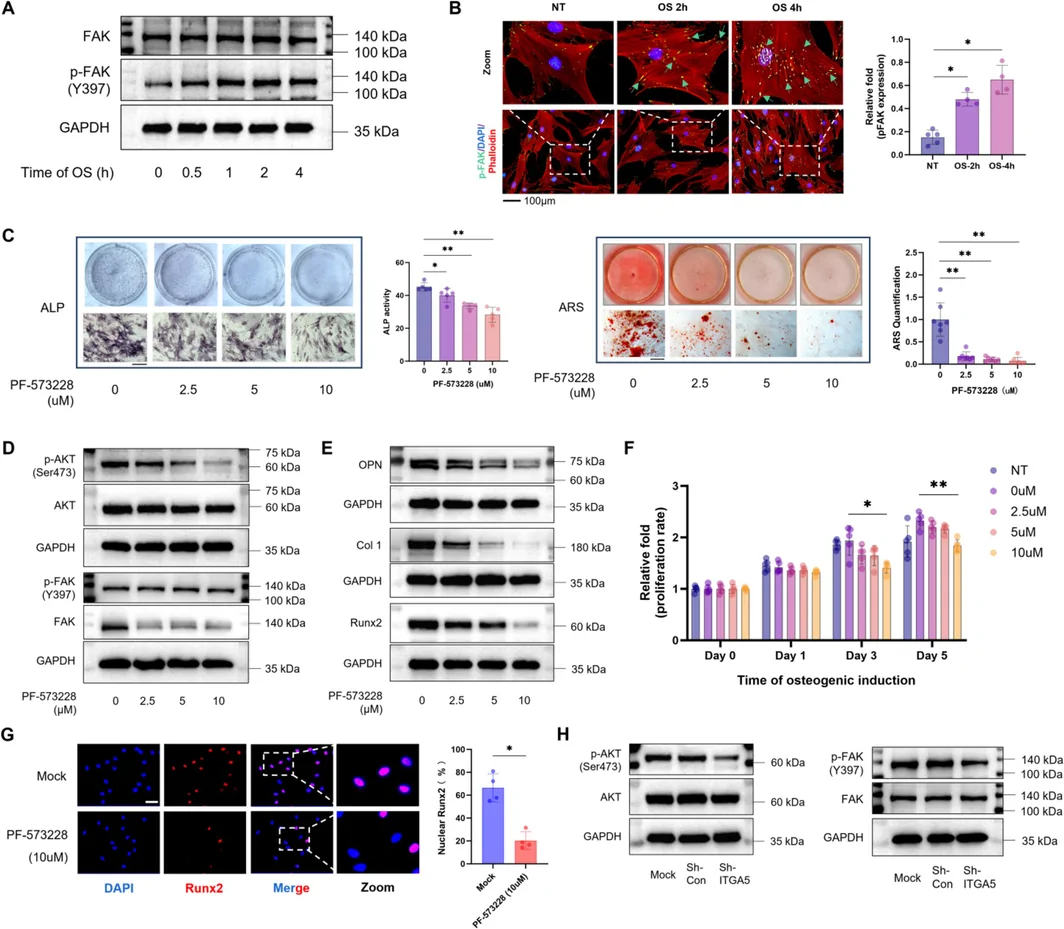
AS‐Fbs promoted osteogenic differentiation via FAK‐phosphorylation following activation of ITGA5. (A) Western blots of phospho‐FAK levels for AS‐Fbs during 0–4 h OS. (B) Immunofluenrence images and quantification of expression levels for phospho‐FAK with 0, 2 and 4 h OS. Scale bar: 100 μm. (C) ALP, ARS staining and quantitative assessment of AS‐Fbs with OS and PF‐573228 (concentration, 0‐10 μM) intervention after 7‐ and 21‐day OS, respectively. Scale bar: 300 μm. (D,E) Western blots of expression levels for phospho‐AKT (Ser473), phospho‐FAK (Y397), OPN, Col1 and Runx2 in AS‐Fbs treated with PF‐573228 (concentration, 0‐10 μM). (F) The CCK assay showed the cell proliferation of AS‐Fbs with the intervention of PF‐573228 during the 5‐day osteogenic induction. (G) Immunofluenrence images and quantification for nuclear translocation of Runx2 rate in AS‐Fbs with/without PF‐573228 intervention (10 μM). Scale bar: 100 μm. (H) Western blots for phospho‐AKT (Ser473) and phospho‐FAK (Y397) levels in AS‐Fbs with PBS, Sh‐control and Sh‐ITGA5 transfection. **p < 0.01, *p < 0.05. ALP, alkaline phosphatase; ARS, alizarin red staining; AS, ankylosing spondylitis; Col 1, collagen I; FAK: Focal adhesion kinase; Fbs, fibroblasts; ITGA5: Integrin alpha 5; OPN, osteopontin; OS, osteogenic stimulation; Runx2, runt‐related transcription factor 2.

### 3.7 Validation of Osteogenic Capacity for AS‐Fbs In Vivo

To validate the osteogenic capacity of AS‐Fbs and simulate the process of pathological osteogenesis in vivo, isolated Fbs treated with TNF‐α and/or Ly294002 were transplanted into the subcutaneous tissue of nude mice after osteogenic induction, respectively (Four conditions were tested: OS, OS+TNF, OS+Ly and OS+TNF+Ly) (Figure 7A). The histological appearance, osteogenic markers expression, and the mineralised matrix detected by μCT indicated the formation of the bone‐like tissue following 2–3 month transplantation (Figure 7B–E). Moreover, with the stimulation of low concentration TNF‐α, the harvested tissue exhibited a relatively larger volume and more distinct osteogenic characteristics than that without inflammatory intervention after 2 months of transplantation (Figures 7B and S6A). Immunofluenrence staining of representative osteogenic markers was also performed to clarify the nature of the tissue and degree of osteogenesis. The results revealed higher expression levels of Col 1, Runx2, and OCN in the TNF‐treated group than the group without TNF‐α pre‐treatment (Figure 7C,D). To further elucidate the role of PI3K/AKT signals in inducing ectopic bone formation in vivo, Ly294002 was applied for cells in advance of transplant. Results of μCT and histological staining showed reduced bone‐like tissue formation after inhibitor intervention following 2 months of transplantation (Figure 7E,F). Immunofluorescence analysis also revealed that inhibition of the core pathway for Fbs diminished the osteogenic potential of ectopic tissues (Figure 7G,H). The ALP activity staining for the tissue sections in the 4 conditions (OS, OS+TNF‐α, OS+Ly294002, OS+TNF‐α+Ly294002) also provided strong, complementary evidence to support our conclusion of bone‐like tissue formation (Figure S6B). These results confirmed the osteogenic capacity of AS‐Fbs in vivo and the key role of activation of the PI3K/AKT pathway for pathological osteogenesis.

**FIGURE 7:**
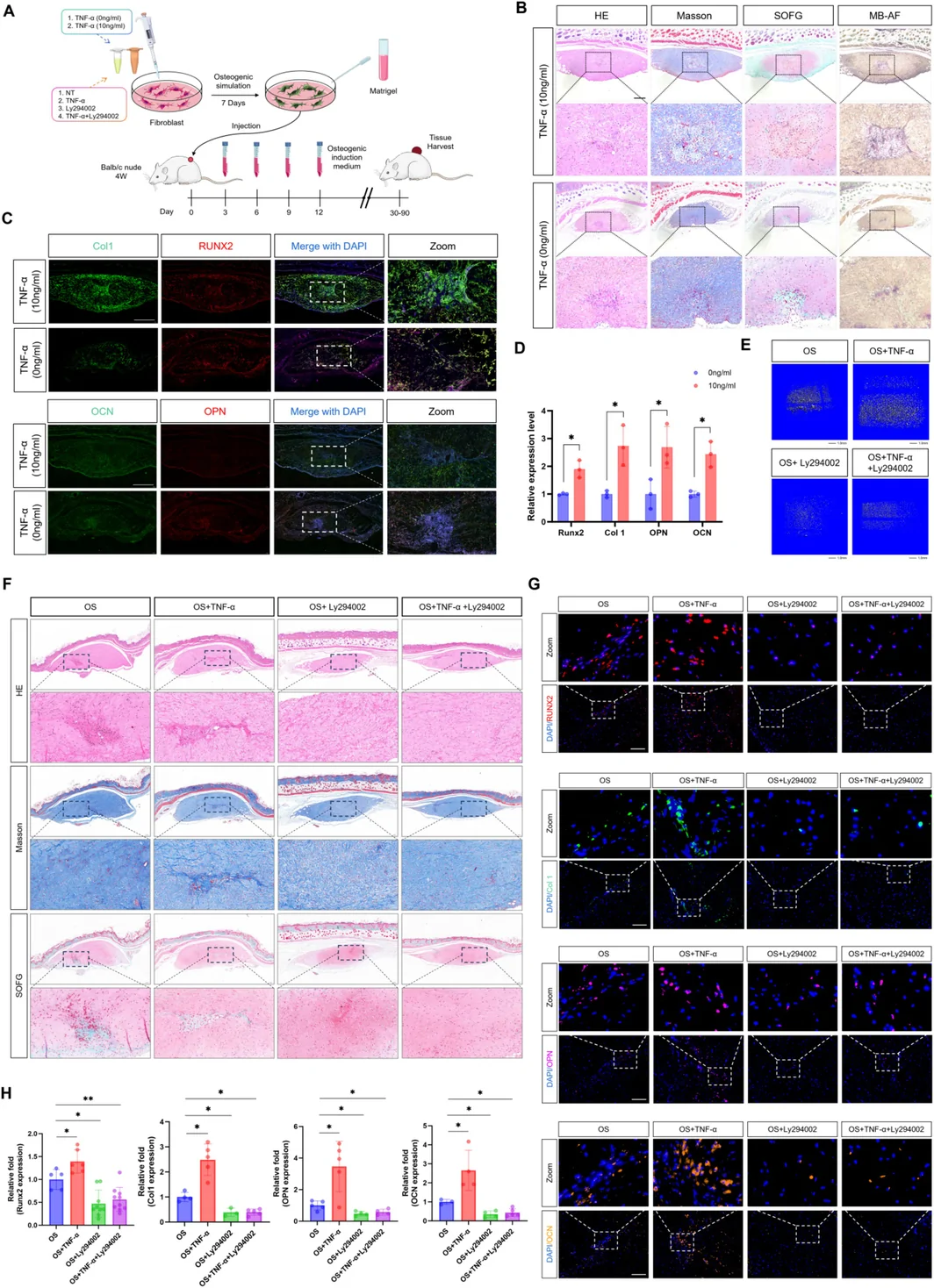
Validation of pathological osteogenesis for AS‐Fbs in vivo. (A) An illustration of the AS‐Fbs subcutaneous transplantation model for ectopic ossification in nude mice. (B) H&E, Masson, SOFG and MB‐AF staining of ectopic new bone formation. MB‐AF staining confirmed osteoid formation in the grafts of both groups. Relative to the control group, the inflammation‐stimulated group presented a higher proportion of osteoblasts within the grafts and more newly mineralised bone in the central region. Scale bar: 200 μm. (C, D) Immunofluenrence images and quantitative analysis of osteogenic markers in subcutaneous harvested tissue. Scale bar: 400 μm. (E) μCT of ectopic new bone formation. Scale bar: 1 mm. (F) H&E, Masson and SOFG staining of new bone formation. Scale bar: 200 μm. (G, H) Immunofluenrence images and quantitative analysis of Runx2, Col 1, OPN and OCN for harvested tissues transplanted with AS‐Fbs stimulated by TNF‐α and Ly294002. **p < 0.01, *p < 0.05. Scale bar: 200 μm. AS, ankylosing spondylitis; Col 1, collagen I; Fbs, fibroblasts; Runx2, runt‐related transcription factor 2; H&E, haematoxylin–eosin; MB‐AF, methylene blue‐fuchsin acid; OCN, osteocalcin; OPN, osteopontin; OS, osteogenic stimulation; SOFG, Safranin O‐Fast Green.

## 4 Discussion

In the present study, we have centered our investigations on the clinical challenge regarding the discrepancy between effective anti‐inflammatory therapy and insufficient inhibition of HO process. The predominant findings encompassed three main aspects: (1) AS‐Fbs derived from in situ ligament tissues exhibited greater osteogenic transdifferentiation capacity than those from healthy individuals and might be involved in the aetiology of ectopic ossification, yet they were frequently overlooked in previous research focuses; (2) persistent low‐concentration TNF‐α stimulation induced enhanced pathological osteogenesis; this observation might align with the residual inflammatory micro‐environment (attributed to incomplete inflammation suppression) of AS patients following periodic application of a single anti‐inflammatory targeted drug; and (3) identifying a novel axial signalling pathway (ECM/ITGA5/FAK/AKT) mediating osteogenic transdifferentiation, thereby providing therapeutic targets for combination with anti‐inflammatory therapy and addressing the aforementioned clinical discrepancy.

The primary finding of the present study was that AS‐Fbs, as ubiquitous mesenchymal cells, played a pivotal role in osteogenesis by maintaining tissue homeostasis, synthesising and remodelling extracellular matrix (ECM) [16, 17, 37]. Originating from mesenchymal cells, these cells exhibited remarkable plasticity, enabling their phenotypic transition in response to microenvironmental signals [15, 38]. In AS, emerging studies have turned their attention to Fb transdifferentiation in the context of pathological ossification. Zeng et al. reported that osteogenic differentiation of AS‐Fbs could be significantly activated and promoted by histone deacetylase 1 (HDAC1) [38]. Furthermore, He et al. demonstrated that osteogenic effect and transdifferentiation of AS‐Fbs could be induced by aberrant up‐regulation of growth differentiation factor 15 (GDF‐15) [18]. In addition, numerous studies have also been conducted on the osteoblastic transdifferentiation of Fb on cells extracted from the periodontal ligament [39, 40, 41]. Consistently, our findings also indicated that Fbs derived from AS spinal ligaments possessed superior osteogenic capacity and transdifferentiational potential. In light of our findings, the previously overlooked osteogenic potential of Fbs in ligamentous tissues deserved more attention for intervention and treatment of ectopic ossification caused by AS.

Another crucial finding in the current study was that a low‐concentration TNF‐α could sustainably enhance the osteogenic differentiation of AS‐Fbs and promote ectopic ossification more than that of relative high‐level inflammatory stimulation. Previous studies have shown that the relationship between inflammation and ectopic ossification progression in AS still remained inconclusive [3, 5, 42, 43, 44, 45]. Despite the fact that multiple clinical trials have confirmed that targeted anti‐inflammatory drugs could precisely and effectively control the inflammation progression in AS, pathological new bone formation persisted after repeated administration of these agents, which proved to be a key challenge in clinical treatment. In conjunction with our results, this phenomenon could be clarified from the following aspects: (1) the presence of inflammation might not be the sole initiating factor for heterotopic osteogenesis; instead, it was more likely to act merely as an auxiliary factor in pathological ossification [37]; (2) more importantly, the inflammation secondary to AS was associated with multiple cell types or diverse pathways [5, 7, 46, 47, 48, 49, 50]; thus, following the intermittent or periodic administration of a single anti‐inflammatory agent, a great amount of AS patients might maintain a low‐grade chronic inflammatory micro‐environment within their bodies; in this situation, pathological osteogenic differentiation still continued to progress rapidly with the inflammation stimulation. In addition, a previous study has also demonstrated that extracellular vesicles (EVs) could evade the intervention of anti‐inflammatory drugs by enclosing various pro‐inflammatory factors, and subsequently transported these factors to the ligamentous region to establish a local microinflammatory milieu—this might further represent a potential mechanism contributing to the inability of anti‐inflammatory therapy to effectively inhibit HO [26]. According to the results of immunohistochemical staining for AS spinal ligaments, higher expression levels of TNF‐α and infiltration of inflammatory cells were identified than that of the control group, confirming the aforementioned suppositions. To further validate the osteogenic potential of Fbs and simulate the process of pathological ossification in vivo, isolated and purified cells were transplanted subcutaneously to the nude mice. Notably, we found that the harvested tissue derived from Fbs stimulated with low‐concentration TNF‐α could generate larger tissue masses and exhibit elevated expression of osteogenic markers relative to control cells without prior inflammatory cytokine priming, which appeared to be more likely to form subcutaneous ectopic bony tissue. Taken together, these findings underscored the positive regulatory role of the low‐grade inflammatory microenvironment in the occurrence of pathological osteogenesis and provided partial evidence for the occult HO progression in the context of anti‐inflammatory therapy. Besides, in order to precisely simulate the internal pathogenic conditions, all the cells for subsequent molecular mechanism experiments were stimulated with low‐concentration TNF‐α (10 ng/mL).

Lastly, after clarifying the phenomenon of HO evasion in AS‐Fbs following anti‐inflammatory therapy, it was of particular importance to focus on extra factors beyond inflammation for the therapeutic targets identification. On one hand, we verified the typical pathway (PI3K/AKT) as the downstream molecular signalling involved in pathological osteogenesis [33, 51, 52, 53]. The administration of specific PI3K/AKT inhibitor Ly294002 could result in a significant suppressive influence on pathological new bone formation in vitro and in vivo experiments. On the other hand, we revealed that the interaction of ECM proteins and ITGA5 might exert a pivotal role in pathological osteogenesis secondary to AS [54, 55]. Notably, ITGA5, responsible for the exchange and transduction of intracellular and extracellular signals [56, 57, 58], was found to be a crucial role in pathological ossification. Following the administration of Sh‐ITGA5, the significant reduction of expression levels for FAK and AKT phosphorylation was detected, so as to the osteogenic signs suppression, which elucidate the detailed critical molecular signalling. Taken together, based on our results, the confirmation of ECM/ITGA5/FAK/AKT axial pathway could provide multiple novel therapeutic targets for pathological osteogenesis intervention of AS‐Fbs, which might be combined with anti‐inflammatory treatment for HO progression treatment.

Notably, in terms of the subcutaneous ectopic bone formation model in nude mice, it merely reflected the cell‐autonomous osteogenic potential of ligament‐derived Fbs instead of the physiological mechanical stress found in spinal entheses. However, it was worth noting that the ITGA5/FAK axis in the present study was well‐known mechanotransducers, indicating the potential biomechanics‐related aetiology for pathological ossification caused by Fbs. Actually, in the clinical AS, the synergistic effects of residual low‐grade inflammatory micro‐environment reported by our study and abnormal physiological mechanical stress should be emphasised and explicated, including two aspects: (1) Chronic inflammatory infiltration in the spinal entheseal regions triggered the development of heterotopic ossification and enhanced the responsiveness of local Fbs to inflammatory stimuli; on this basis, continuous or discontinuous osteophytes formed on the anterior aspect of the vertebral bodies; (2) Once continuous osteophytes have formed, the regions bearing discontinuous osteophytes were subjected to persistent micro‐motion during daily activities and, owing to the overall rigidity of the spine, readily became the centre of stress concentration. Following sustained exposure to excessive abnormal mechanical forces, local cells might undergo pathological osteogenesis via mechanotransduction (e.g., ITGA5/FAK, YAP/TAZ signalling pathways). Furthermore, stress‐concentrated regions were predisposed to the formation of pseudarthrosis, in which rapid proliferation of fibrous tissue secondary to Fbs ultimately transformed into osseous tissue, thereby accelerating the ectopic ossification.

Nonetheless, the results of the present study should be interpreted in terms of its several limitations. Firstly, most of the patients selected for sampling were in the middle‐to‐late stages of the disease, leading to the degree of ossification varying among the patients, which might result in different states of the isolated cells. Secondly, although multiple animal models (including Collagen Antibody‐Induced Arthritis [CAIA], Proteoglycan‐Induced Spondylitis [PGIS], spontaneous arthritis model of DBA/1) were reported to present with similar pathological features to AS, the primary site and progression process of pathological ossification were not identical to the disease. Thus, a more representative animal model was warranted for further investigation. Thirdly, whether any other pathway contributes to the promotion of osteogenic proteins following ITGA5 activation needs to be explored in the future. Fourthly, in the present study, the normal control group consisted of uncalcified ligaments from patients with ‘thoracolumbar fractures’. Notably, acute thoracolumbar fracture was frequently accompanied by prominent local inflammatory activation, with rapid up‐regulation of TNF‐α and other pro‐inflammatory mediators. Such a trauma‐primed micro‐environment could theoretically modify the basal phenotype of ligament‐derived Fbs, thereby biasing the comparison between the control and disease groups. Nonetheless, several considerations that may mitigate this concern were listed as follows [59, 60]: (1) The post‐fracture inflammatory response was time‐limited. Previous studies demonstrated that inflammatory cytokines rapidly increased after bone injury, peaked at 24–48 h, and gradually returned to baseline after 48 h. In our study, control samples were collected during definitive surgical fixation, typically 3–5 days after acute trauma, so local inflammation at the injury site was theoretically near baseline. Consistent with this, Figure 3J showed significantly lower TNF‐α expression in control ligaments relative to AS group; (2) Moreover, some patients received preoperative NSAIDs for severe pain, which might reduce local inflammatory cytokine expression.; (3) The ligament‐derived Fbs subjected to phenotypic and functional analyses were isolated, expanded, and cultured for multiple passages under standard conditions prior to assessment. This in vitro expansion process effectively diluted and eliminated the transient in vivo inflammatory micro‐environment existing at the time point of tissue harvest, further minimising residual trauma‐related inflammatory effects on the measured outcomes. Fifthly, the 10 ng/mL dose of TNF‐α was defined as representing the ‘residual inflammatory microenvironment’ post‐therapy; however, it must be admitted that the aforementioned dose could not equate to the true in vivo concentration and directly quantifying the TNF‐α concentration within the target ligament tissue remained technically challenging for several reasons [40, 61, 62, 63, 64, 65]: (1) The coexistence of soluble (sTNF‐α) and transmembrane (mTNF‐α) forms in tissue fundamentally complicated local cytokine quantification, because most available antibodies recognise shared epitopes and could not distinguish between the two forms; these antibodies also frequently binded sTNF‐α (already complexed with its receptors), producing false‐positive signals that overestimate mTNF‐α, while low mTNF‐α surface expression and the short half‐life of sTNF‐α (approximately 15 min) further hinder reliable measurement; (2) the dense, fibrous nature of ligament tissue, together with marked spatial heterogeneity, complicated reproducible extraction and quantification; (3) circulating TNF‐α levels could not reliably reflect local tissue concentrations and the serum TNF‐α was generally in the low‐pg/mL range (instead of ng/mL). Consequently, there was currently a paucity of published data establishing a definitive reference range for TNF‐α within the ligamentous micro‐environment of AS patients. In spite of this, our histopathological observations independently supported the biological plausibility of a persistent local inflammatory micro‐environment in this surgical cohort (regularly receiving anti‐inflammatory medication before surgery). Given these constraints, the selection of 10 ng/mL represented a pragmatic and biologically grounded approach and functionally validated stimulus to model a pro‐inflammatory microenvironment in a controlled in vitro system. To address this issue more rigorously in future work, tissue homogenisation combined with sensitive ELISA‐based quantification of inflammatory cytokines, microdialysis‐based sampling of peri‐ligamentous interstitial fluid would be tried and performed, thereby further validating the in vitro dose adopted here.

## 5 Conclusions

We identified the transdifferentiational potential and significantly enhanced osteogenic capacity of AS‐Fbs than that of normal populations. Besides, by confirming the positive effect of low‐concentration TNF‐α on aberrant osteogenesis of AS‐Fbs, the potential causes underlying persistent HO progression following anti‐inflammatory therapy were elucidated. Additionally, the activation of ECM/ITGA5/FAK/AKT axial pathways acted as a crucial role in the pathological osteogenesis, which provided novel therapeutic strategies for ectopic ossification via suppression of these signals.

## Author Contributions

**Mu Qiao:** conceptualisation, investigation, data curation, methodology, validation, writing – original draft, writing – review and editing. **Kai‐Yang Wang:** conceptualisation, methodology, funding acquisition, supervision, project administration, writing – review and editing. **Chen‐Yu Song:** investigation, methodology, formal analysis, writing – review and editing. **Jing‐Shun Lu:** investigation, methodology, formal analysis, writing – review and editing. **Jiu‐Kun Li:** investigation, formal analysis, writing – review and editing. **Yong Qiu:** funding acquisition, project administration, writing – review and editing. **Bang‐Ping Qian:** conceptualisation, funding acquisition, project administration, supervision, writing – review and editing. **Ze‐Zhang Zhu:** conceptualisation, formal analysis, validation, project administration, supervision, writing – review and editing.

## Funding

This study was financially supported by the following sources in China, including: Jiangsu Provincial Medical Talent Program (ZDRCA2016068); Jiangsu Provincial Key Medical Center (YXZXA2016009); China Postdoctoral Science Foundation (2025M782167); Shanghai Post‐doctoral Excellence Program (2025461) and Postdoctoral Fellowship Program of China Postdoctoral Science Foundation (GZC20261229).

## Ethics Statement

Ethics Committee of Nanjing drum tower Hospital agreed to this experiment (2024‐787‐03). All the animal experiments were reviewed and approved by the Laboratory Animal Ethics Committee of Nanjing Hospital Affiliated to Nanjing Medical University and Nanjing Drum Tower Hospital, Affiliated Hospital of Medical School, Nanjing University (DWSY‐23035339). The raw data used or analysed in the study were available from the corresponding author on reasonable request.

## Consent

Informed consent was obtained from all patients for being included in the study.

## Conflicts of Interest

The authors declare no conflicts of interest.

## Supporting information


**Figure S1:** Quantitative analysis of western blots and immunofluorescence staining for intergroup differences in the osteogenic capacity of Fbs. (A) comparison of specific markers (Col 1, Runx2, OPN and OCN) during the process of osteogenic trans‐differentiation between AS‐Fbs and control‐Fbs. (B, C) FAP expression before and after the osteogenic induction in AS‐Fbs. (D) osteocytes‐derived secreted proteins (DMP1, MEPE and SOST) in AS group at day 0 (D0) and day 21 (D21) following osteogenic induction. ****p < 0.0001, **p < 0.01, *p < 0.05. AS, ankylosing spondylitis; Col 1, collagen I; DMP1, dentine matrix acidic phosphoprotein 1; FAP, fibroblast activation protein; Fbs, fibroblasts; MEPE, matrix extracellular phosphoglycoprotein; NT, Non‐treatment; OCN, osteocalcin; OPN, osteopontin; OS, osteogenic stimulation; Runx2, runt‐related transcription factor 2; SOST, sclerostin.
**Figure S2:** Effects of various concentrations of TNF‐α on the osteogenic differentiation of control‐Fbs and quantitative analysis of western blots. (A) ARS staining and quantification of pathologic ossification for control‐Fbs with various dose of TNF‐α stimulation (0–100 ng/mL) after 21‐day OS. (B) ALP staining and quantification of pathologic ossification for control‐Fbs with various dose of TNF‐α stimulation (0–100 ng/mL) after 7‐day OS. (C) quantitative evaluation of expression levels for osteogenic markers (Runx2/Col1) in western blots after OS for AS‐Fbs with low‐to‐high concentration of TNF‐α treatment. (D) quantification of immunochemistry staining for TNF‐α in AS and control spinal ligaments. *p < 0.05. ALP, alkaline phosphatase; ARS, alizarin red; AS, ankylosing spondylitis; Col 1, collagen I; Fbs, fibroblasts; OS, osteogenic stimulation; Runx2, runt‐related transcription factor 2; TNF‐α, tumour necrosis factor‐alpha.
**Figure S3:** Quantitative analysis of western blots for the validation of activation for PI3K/AKT pathway. (A) expression levels of phospho‐AKT (Ser473) during the osteogenic induction. (B, C) expression levels of phospho‐AKT (Ser473), OCN, Col 1, OPN and Runx2 in AS‐Fbs after TNF‐α and Ly294002 intervention. (D, E) ARS, ALP staining and relevant quantitative assessment of AS‐Fbs treated with TNF‐α and/or MK‐2206. Scale bar: 10 μm. (F) Western blots evaluation of the expression levels for phospho‐AKT (Ser473), Col 1 and Runx2 in AS‐Fbs after TNF‐α and MK‐2206 intervention. ***p < 0.01, **p < 0.01, *p < 0.05. ALP, alkaline phosphatase; ARS, alizarin red; AS, ankylosing spondylitis; Col 1, collagen I; Fbs, fibroblasts; OCN, osteocalcin; OPN, osteopontin; Runx2, runt‐related transcription factor 2.
**Figure S4:** Abnormal osteogenic differentiation of AS‐Fbs depended on the activation of ITGA5 mediated by ECM. (A, B) GO terms and KEGG‐enriched analysis of differentially expressed genes in AS and control‐Fbs. (C) quantitative analysis of western blots showed the OS time‐depended expression levels of THBS1 and FBLN1. (D) quantitative analysis of western blots for THBS1 and FBLN1 between AS and control‐Fbs at 2‐day osteogenic stimulation. (E) quantitative analysis of immunohistochemical staining for ITGA5 in AS and control spinal ligament tissues. (F) quantitative evaluation of western blots for ITGA5 in AS and control‐Fbs. (G) quantitative assessment of western blots for ITGA5 expression levels for AS‐Fbs with 0 to 3 days OS. (H) quantitative analysis of western blots for expression levels of ITGA5 in AS‐Fbs with stimulation of THBS1 and FBLN1 (0–100 ng/mL) for 4 h. (I, J) quantitative analysis of western blots for ITGA5, Col1, Runx2, OPN and OCN levels after Sh‐ITGA5 transfection. (K) Western blots and quantitative analysis showed higher expression level of THBS1, FBLN1 and ITGA5 in low TNF‐α concentration group (10 ng/mL) than that of the other two groups with inflammatory stimulation of 0 and 100 ng/mL. ****p < 0.0001, ***p < 0.001, **p < 0.01, *p < 0.05. AS, ankylosing spondylitis; Col 1, collagen I; Ctrl, control; FBLN1, fibulin 1; Fbs, fibroblasts; ITGA5, integrin alpha 5; OCN, osteocalcin; OPN, osteopontin; OS, osteogenic stimulation; Runx2, runt‐related transcription factor 2; THBS1, thrombospondin 1.
**Figure S5:** Quantitative analysis of western blots for the validation of activation for ITGA5/FAK/AKT signalling axis. (A) expression levels of phospho‐FAK for AS‐Fbs during 0–4 h OS. (B, C) expression levels for phospho‐AKT (Ser473), phospho‐FAK (Y397), OPN, Col1 and Runx2 in AS‐Fbs treated with PF‐573228 (concentration, 0‐10 μM). (D) phospho‐AKT (Ser473) and phospho‐FAK (Y397) levels in AS‐Fbs with PBS, Sh‐control and Sh‐ITGA5 transfection. *p < 0.05. AS, ankylosing spondylitis; Col 1, collagen I; FAK, focal adhesion kinase; Fbs, fibroblasts; ITGA5, integrin alpha 5; OPN, osteopontin; OS, osteogenic stimulation; Runx2, runt‐related transcription factor 2.
**Figure S6:** Validation of the bone‐like tissue formation. (A) The relative cross‐sectional area of the harvest tissues for inflammatory intervention and control group. (B) The ALP activity staining and quantification for the harvest tissue in four conditions. Scale bar: 200 μm. *p < 0.05. OS, osteogenic stimulation.

## Data Availability

The data that support the findings of this study are available from the corresponding author upon reasonable request.
