# Supplementary material for: Pathological Ossification of Spinal Ligament‐Derived Fibroblasts in an Inflammatory Micro‐Environment via Activation of the ECM/ITGA5/FAK/AKT Pathway in Ankylosing Spondylitis
Source: Cell Prolif. 2026 Nov 6:e70299. Online ahead of print. doi: 10.1111/cpr.70299 (PMC13743320; doi:10.1111/cpr.70299)
Supplement: Supplementary file 1 [file CPR-9999-e70299-s001.docx]

**Supplementary figures**

**Figure S1.** Quantitative analysis of western blots and immunofluorescence staining for intergroup differences in the osteogenic capacity of Fbs. **A)** comparison of specific markers (Col 1, Runx2, OPN and OCN) during the process of osteogenic trans-differentiation between AS-Fbs and control-Fbs. **B,C)** FAP expression before and after the osteogenic induction in AS-Fbs. **D)** osteocytes-derived secreted proteins (DMP1, MEPE and SOST) in AS group at day 0 (D0) and day 21 (D21) following osteogenic induction. *****P*<0.0001, ***P*<0.01, **P*<0.05. AS, ankylosing spondylitis; Fbs, fibroblasts; Runx2, runt-related transcription factor 2; Col 1, collagen I; OPN, osteopontin; OCN, osteocalcin; NT, Non-treatment; OS, osteogenic stimulation; FAP, fibroblast activation protein; DMP1, dentin matrix acidic phosphoprotein 1; MEPE, matrix extracellular phosphoglycoprotein; SOST, sclerostin.

**Figure S2.** Effects of various concentrations of TNF-α on the osteogenic differentiation of control-Fbs and quantitative analysis of western blots. **A)** ARS staining and quantification of pathologic ossification for control-Fbs with various dose of TNF-α stimulation (0-100ng/ml) after 21-day OS. **B)** ALP staining and quantification of pathologic ossification for control-Fbs with various dose of TNF-α stimulation (0-100ng/ml) after 7-day OS. **C)** quantitative evaluation of expression levels for osteogenic markers (Runx2/Col1) in western blots after OS for AS-Fbs with low-to-high concentration of TNF-α treatment. **D)** quantification of immunochemistry staining for TNF-α in AS and control spinal ligaments. **P*<0.05. TNF-α, tumor necrosis factor-alpha; AS, ankylosing spondylitis; Fbs, fibroblasts; ARS, alizarin red; OS, osteogenic stimulation; ALP, alkaline phosphatase; Runx2, runt-related transcription factor 2; Col 1, collagen I.

**Figure S3.** quantitative analysis of western blots for the validation of activation for PI3K/AKT pathway. **A)** expression levels of phospho-AKT (Ser473) during the osteogenic induction. **B,C)** expression levels of phospho-AKT (Ser473), OCN, Col 1, OPN and Runx2 in AS-Fbs after TNF-α and Ly294002 intervention. **D,E)** ARS, ALP staining and relevant quantitative assessment of AS-Fbs treated with TNF-α and/or MK-2206. Scale bar: 10μm. **F)** Western blots evaluation of the expression levels for phospho-AKT (Ser473), Col 1 and Runx2 in AS-Fbs after TNF-α and MK-2206 intervention. ****P*<0.01, ***P*<0.01, **P*<0.05. AS, ankylosing spondylitis; Fbs, fibroblasts; OCN, osteocalcin; Col 1, collagen I; OPN, osteopontin; Runx2, runt-related transcription factor 2; ARS, alizarin red; ALP, alkaline phosphatase.

**Figure S4.** Abnormal osteogenic differentiation of AS-Fbs depended on the activation of ITGA5 mediated by ECM. **A,B)** GO terms and KEGG-enriched analysis of differentially expressed genes in AS and control-Fbs. **C)** quantitative analysis of western blots showed the OS time-depended expression levels of THBS1 and FBLN1. **D)** quantitative analysis of western blots for THBS1 and FBLN1 between AS and control-Fbs at 2-day osteogenic stimulation. **E)** quantitative analysis of immunohistochemical staining for ITGA5 in AS and control spinal ligament tissues. **F)** quantitative evaluation of western blots for ITGA5 in AS and control-Fbs. **G)** quantitative assessment of western blots for ITGA5 expression levels for AS-Fbs with 0 to 3 days OS. **H)** quantitative analysis of western blots for expression levels of ITGA5 in AS-Fbs with stimulation of THBS1 and FBLN1 (0-100ng/mL) for 4 hours. **I,J)** quantitative analysis of western blots for ITGA5, Col1, Runx2, OPN and OCN levels after Sh-ITGA5 transfection. **K)** Western blots and quantitative analysis showed higher expression level of THBS1, FBLN1 and ITGA5 in low TNF-α concentration group (10ng/mL) than that of the other two groups with inflammatory stimulation of 0 and 100 ng/mL. *****P*<0.0001, ****P*<0.001, ***P*<0.01, **P*<0.05. AS, ankylosing spondylitis; Fbs, fibroblasts; OS, osteogenic stimulation; ITGA5: integrin alpha 5; THBS1: thrombospondin 1; FBLN1: fibulin 1; Ctrl, control; Col 1, collagen I; Runx2, runt-related transcription factor 2; OPN, osteopontin; OCN, osteocalcin.

**Figure S5.** quantitative analysis of western blots for the validation of activation for ITGA5/FAK/AKT signaling axis. **A)** expression levels of phospho-FAK for AS-Fbs during 0-4 hour OS. **B,C)** expression levels for phospho-AKT (Ser473), phospho-FAK (Y397), OPN, Col1 and Runx2 in AS-Fbs treated with PF-573228 (concentration, 0-10μM). **D)** phospho-AKT (Ser473) and phospho-FAK (Y397) levels in AS-Fbs with PBS, Sh-control and Sh-ITGA5 transfection. **P*<0.05. FAK: focal adhesion kinase; AS, ankylosing spondylitis; Fbs, fibroblasts; OS, osteogenic stimulation; OPN, osteopontin; Col 1, collagen I; Runx2, runt-related transcription factor 2; ITGA5: integrin alpha 5.

**Figure S6.** Validation of the bone-like tissue formation. **A)** The relative cross-sectional area of the harvest tissues for inflammatory intervention and control group. **B)** The ALP activity staining and quantification for the harvest tissue in four conditions. Scale bar: 200μm. **P*<0.05. OS, osteogenic stimulation.
